# Neutronics Simulations for DEMO Diagnostics

**DOI:** 10.3390/s23115104

**Published:** 2023-05-26

**Authors:** Raul Luís, Yohanes Nietiadi, Antonio Quercia, Alberto Vale, Jorge Belo, António Silva, Bruno Gonçalves, Artur Malaquias, Andrei Gusarov, Federico Caruggi, Enrico Perelli Cippo, Maryna Chernyshova, Barbara Bienkowska, Wolfgang Biel

**Affiliations:** 1 Instituto de Plasmas e Fusão Nuclear, Instituto Superior Técnico, Universidade de Lisboa, Av. Rovisco Pais 1, 1049-001 Lisbon, Portugal; ynietiadi@ipfn.tecnico.ulisboa.pt (Y.N.); avale@ipfn.tecnico.ulisboa.pt (A.V.); jbelo@ipfn.tecnico.ulisboa.pt (J.B.); silva@ipfn.tecnico.ulisboa.pt (A.S.); bruno@ipfn.tecnico.ulisboa.pt (B.G.); artur.malaquias@ipfn.tecnico.ulisboa.pt (A.M.); 2 DIETI/Consorzio CREATE, Università Federico II, Via Claudio 21, 80125 Napoli, Italy; antonio.quercia@unina.it; 3 SCK CEN Belgian Nuclear Research Center, 2400 Mol, Belgium; andrei.goussarov@sckcen.be; 4 Institute for Plasma Science and Technology, National Research Council, 20125 Milan, Italy; f.caruggi@campus.unimib.it (F.C.); enrico.perellicippo@istp.cnr.it (E.P.C.); 5 Institute of Plasma Physics and Laser Microfusion, Hery 23, 01-497 Warsaw, Poland; maryna.chernyshova@ifpilm.pl (M.C.); barbara.bienkowska@ifpilm.pl (B.B.); 6 Institute of Energy and Climate Research, Forschungszentrum Jülich GmbH, 52428 Jülich, Germany; w.biel@fz-juelich.de

**Keywords:** neutronics, diagnostics, tokamaks, DEMO, nuclear fusion, MCNP

## Abstract

One of the main challenges in the development of a plasma diagnostic and control system for DEMO is the need to cope with unprecedented radiation levels in a tokamak during long operation periods. A list of diagnostics required for plasma control has been developed during the pre-conceptual design phase. Different approaches are proposed for the integration of these diagnostics in DEMO: in equatorial and upper ports, in the divertor cassette, on the inner and outer surfaces of the vacuum vessel and in diagnostic slim cassettes, a modular approach developed for diagnostics requiring access to the plasma from several poloidal positions. According to each integration approach, diagnostics will be exposed to different radiation levels, with a considerable impact on their design. This paper provides a broad overview of the radiation environment that diagnostics in DEMO are expected to face. Using the water-cooled lithium lead blanket configuration as a reference, neutronics simulations were performed for pre-conceptual designs of in-vessel, ex-vessel and equatorial port diagnostics representative of each integration approach. Flux and nuclear load calculations are provided for several sub-systems, along with estimations of radiation streaming to the ex-vessel for alternative design configurations. The results can be used as a reference by diagnostic designers.

## 1. Introduction

One of the main challenges in the development of a plasma diagnostic and control (D&C) system for the demonstration fusion reactor (DEMO) is the need to cope with unprecedented radiation levels in a tokamak during long operation periods. Projected to operate with a fusion power of 2 GW, the DEMO plasma will produce 14 MeV neutrons from deuterium–tritium (D-T) reactions at an approximate rate of 7 × 10^20^ n s^−1^ [1]. Although this results in neutron fluxes in the first wall that are not significantly increased when compared to ITER, the longer pulses in DEMO will lead to higher fluences and displacements per atom (dpa) in the plasma-facing materials [2]. Presently, DEMO operation is scheduled in two phases: a first phase with a “starter” blanket, designed to withstand up to 20 dpa in the first wall steel, and a second phase after blanket replacement, with blankets designed for a higher limit of 50 dpa. ITER plasma-facing components, for comparison, will remain below 4 dpa [3]. The development of materials that can cope with loads one order of magnitude higher than those expected for ITER is one of the main challenges towards the realization of DEMO [4].

In DEMO, the design of a D&C system is the task of the Work Package Diagnostic and Control (WPDC). The aim of this project is to design a D&C system with high reliability and accuracy that allows safe operation of the plasma near its operational limits, to maximize the power output [5]. Based on constraints which go far beyond the case of ITER, including the harsher radiation environment, the need for compatibility with remote maintenance operations and space limitations dictated by the requirements of first wall integrity and tritium breeding, a list of diagnostics required for plasma control has been developed within WPDC during the pre-conceptual design phase. This list includes [6]:

- Magnetic diagnostics (pickup coils, saddle loops, full-flux loops, diamagnetic loops, Rogowski coils, Hall sensors);
- Faraday sensors;
- Infrared (IR) polarimetry/interferometry;
- Neutron and gamma cameras;
- Microwave (MW) reflectometry;
- Electron cyclotron emission (ECE);
- Divertor thermocurrent measurements;
- Radiated power and soft X-ray intensity;
- X-ray spectroscopy;
- Vacuum ultraviolet spectroscopy (VUV) spectroscopy;
- IR/visible (VIS)/near-UV divertor spectroscopy;
- VIS spectroscopy and thermography of limiters;
- Pellet monitoring;
- Collective Thomson scattering (CTS).

**Figure 1:**
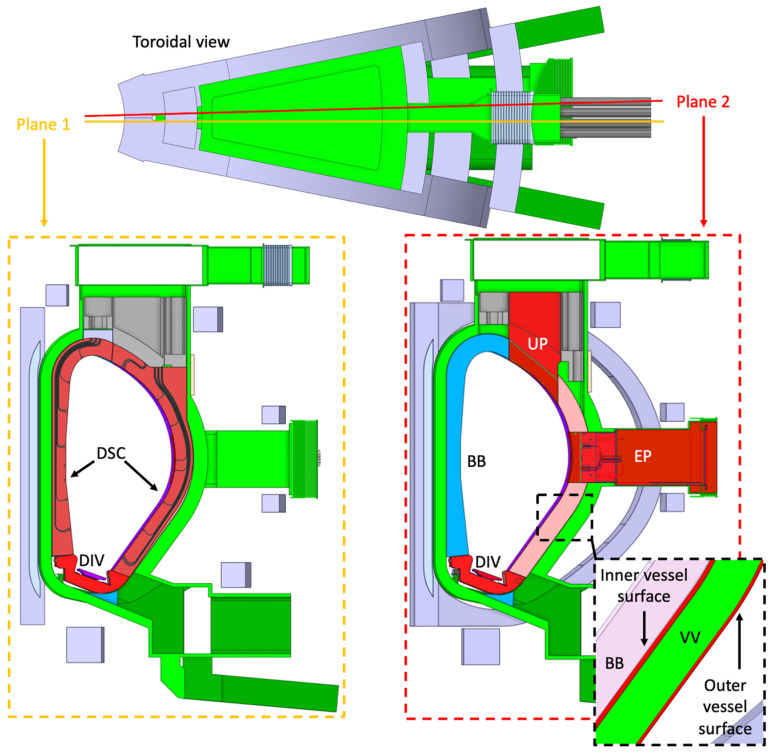
Representation of one DEMO sector and foreseen locations for diagnostics, represented in red (for visualization only—diagnostics will be distributed in different sectors). DSC: Diagnostics Slim Cassette (red). EP: Equatorial Port (red). UP: Upper Port (red). BB: Breeding Blanket (blue—inboard and pink—outboard). DIV: Divertor (red). VV: Vacuum Vessel (green).

Figure 1 illustrates the different approaches followed for the integration of these diagnostics in DEMO (collapsed in a single DEMO sector for easier visualization). Most sub-systems are designed to be integrated in equatorial port (EP) plugs dedicated to diagnostics (five or six EPs are foreseen), in some cases with additional lines of sight in the upper ports (UPs) if there is space reserved for diagnostics in the UPs. These include spectroscopy diagnostics [7], neutron/gamma cameras [8,9], radiated power and soft X-ray intensity [10] and IR polarimetry/interferometry [11], with the eventual addition of collective Thomson scattering [12], still under study. For diagnostics that require access to the plasma from several poloidal positions, such as MW reflectometry [13] and ECE [14], the diagnostics slim cassette (DSC) concept has been developed [15,16,17] as a modular approach compatible with the remote handling operations of the breeding blanket (BB). Thermocurrent measurements are planned to be integrated within the divertor cassette [18], while Faraday sensors are distributed poloidally on the outer surface of the vacuum vessel (VV) [19]. Finally, magnetic sensors are distributed on the inner and outer surfaces of the VV [20,21,22].

According to each integration approach, diagnostics in DEMO will be exposed to different radiation levels. This will have a considerable impact on their design. Moreover, radiation streaming to the ex-vessel, either through diagnostic ducts in the ports or due to inadequate shielding from the DSC or other diagnostic components, shall be minimized, in order to comply with the radiation limits defined for the DEMO plant. These include 0.3–0.5 W/cm^3^ and 2.75 dpa in the VV stainless steel, 50 W/m^3^ in the winding packs of the superconductor coils and 100 μSv/h of dose rate in the port cells 12 days after shutdown [1].

This paper aims to provide estimations of the fluxes and nuclear loads in preliminary designs of diagnostics representative of each integration approach, to be used as a reference for diagnostic designers. Although compliance with all the radiation limits set out for DEMO is beyond the scope of this work, such an evaluation is presented when possible, namely with regard to the nuclear heat loads and dpa in the VV. Previous studies have been published with neutronics simulations for the DSC concept [17,23,24], but with limited results for the full DSC and its impact on the neutron and gamma fluxes in the VV. A work focused on an early design of the divertor survey visible high-resolution spectrometer has also been published, which aimed mainly to assess the loads in the first mirrors and the impact of the number of doglegs in the EP ducts on the radiation streaming to the port cells [25]. The objective now is to extend those simulations to more complex geometries and to include additional diagnostics that were not studied before.

Section 2 provides a description of the simulation methods common to all the analyses presented in the paper. Section 3 is the main body of the paper, presenting the models and results obtained for each set of diagnostics: inner-vessel diagnostics, ex-vessel diagnostics (Faraday sensors) and equatorial port diagnostics. Finally, a summary and discussion of the results are provided in Section 4.

## 2. Simulation Methods

### 2.1. Simulation Workflow

All simulations were performed with the Monte Carlo simulation program MCNP6 [26,27], approved for neutronics simulations in the DEMO project [1]. The JEFF-3.3 [28] and FENDL-3.1d [29] neutron cross-section libraries were used in the simulations. The CAD models were produced or edited with CATIA V5 [30] and simplified for conversion to the MCNP input format using ANSYS SpaceClaim 2021R2 [31]. Conversion was carried out with SuperMC 3.3.0 [32,33] and/or McCad v0.5 [34], depending on the requirements of the conversion, and the MCNP simulations were run in the MARCONI-FUSION high-performance computing cluster [35]. The results were processed using Mathematica 13.0 [36], Python 3.9 [37] and Paraview 5.9 [38]. Neutron fluxes, when presented, follow the VITAMIN-J 175 group structure [39].

### 2.2. DEMO Reference Models

The simulations are grouped into three sections, according to each integration approach:

- Inner-vessel diagnostics (excluding port diagnostics);
  ○ In-vessel magnetics sensors;
  ○ Diagnostics slim cassette (reflectometry);
- Ex-vessel diagnostics;
  ○ Faraday sensors;
- Equatorial port diagnostics;
  ○ Spectroscopy diagnostics;
  ○ Neutron/gamma cameras;
  ○ Radiated power and soft X-ray intensity.

The first group includes the in-vessel diagnostics that are not integrated in the ports. Although the divertor thermocurrent diagnostic was not simulated, the results obtained for magnetics sensors below the divertor allow a first estimate of the fluxes and loads in this region, with the important caveat that the integration studies for the divertor in DEMO are still in a very preliminary phase, with ongoing studies and experiments to define the best configurations and strategies to deal with the very high thermal loads [3,40]. Similarly, since the ECE diagnostic is expected to be integrated in a DSC, the results obtained for reflectometry are representative as a first estimation for ECE components. The second group contains simulations for Faraday sensors, which also allow for a first estimate of the fluxes in the ex-vessel magnetics sensors, to be studied in more detail at a later stage. The third group contains several equatorial port diagnostics. Combined with the results presented in reference [41], it provides a broad perspective for most of the diagnostics projected for the ports, with the exception of IR polarimetry/interferometry, not yet simulated, and collective Thomson scattering, for which no design has been proposed for DEMO yet. Due to the lack of a consolidated design for the upper ports, including updated designs of the blanket pipe modules, shielding materials and other systems that will impact the design of diagnostics, no simulations are presented here for upper port systems, which are expected to contain additional lines of sight for the equatorial port diagnostics listed above. This shortcoming shall be addressed in future works.

The blanket configuration assumed in all the simulations was the water-cooled lithium lead (WCLL) BB. The alternative configuration, helium-cooled pebble bed (HCPB), has not been studied yet. Based on comparisons between the two blankets, it can be anticipated that the fluxes behind the blankets with the HCPB configuration would exceed those obtained here by up to one order of magnitude, or even more [1]. This would mostly impact the results obtained for diagnostics located in the inner and outer surfaces of the VV (magnetics and Faraday sensors). It would also imply a redesign of the DSC with helium cooling, with an obvious impact on the shielding and thermomechanical performance of the DSC.

#### 2.2.1. In-Vessel Diagnostics

**Figure 2:**
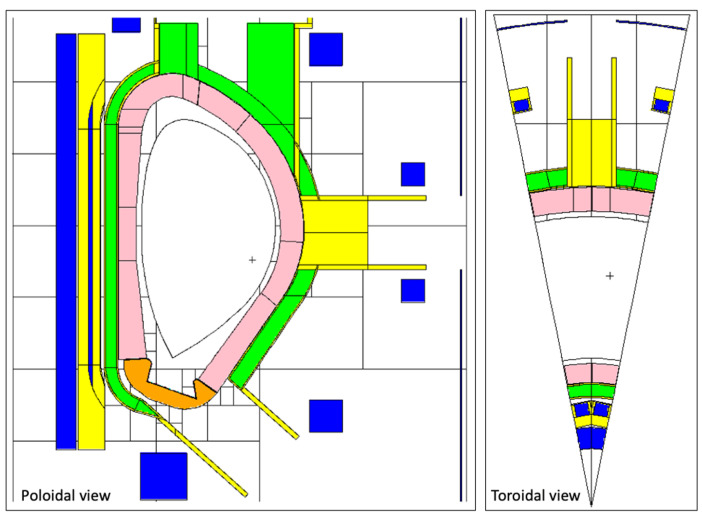
DEMO MCNP reference model used for the simulation of in-vessel diagnostics (excluding port diagnostics). **Left**: Plane y = 10.5 cm. **Right**: Plane z = 0.

The MCNP reference model used for the first group of diagnostics, including the in-vessel diagnostics not integrated in the ports, is represented in Figure 2 [41]. A 22.5-degree model was used, corresponding to a full sector out of the 16 into which DEMO is divided. The blankets of this model were filled with a mixture representative of the WCLL BB, composed of tungsten, EUROFER, water and PbLi [17]. Using a homogenized material in the blankets reduces the complexity of the model, improving the simulation time and allowing for a reasonable first estimate of the fluxes and loads behind the blankets.

#### 2.2.2. Ex-Vessel Diagnostics

**Figure 3:**
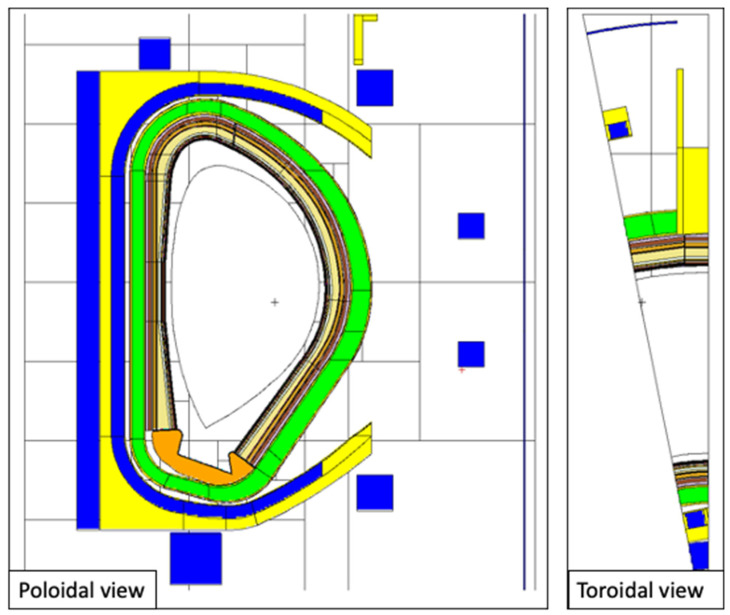
Reference model used in the simulations of the ex-vessel Faraday sensors.

The reference model used for ex-vessel diagnostics (Faraday sensors) is represented in Figure 3 [42]. This is a smaller model when compared to the previous one (11.25° instead of 22.5°), containing a semi-heterogeneous representation of the WCLL blanket which provides good accuracy for ex-vessel simulations while decreasing the simulation times [43].

#### 2.2.3. Equatorial Port Diagnostics

**Figure 4:**
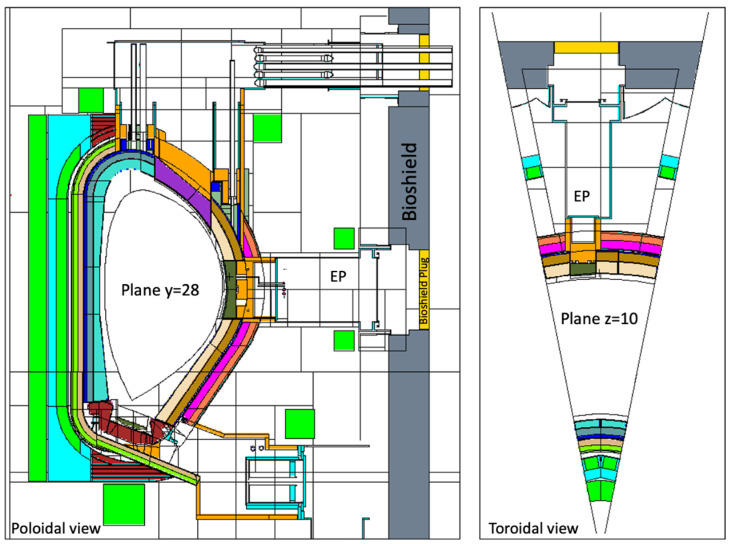
MCNP reference model used in the simulations of equatorial port diagnostics. **Left**: Plane y = 28 cm. **Right**: Plane z = 10 cm.

The reference MCNP model used for equatorial port diagnostics is illustrated in Figure 4 [44]. The model features an upper port, equatorial limiter port and lower pumping port and includes layered representations of two HCPB BBs and one layered representation of the WCLL BB, as described with more detail in reference [45]. For these studies, the WCLL option was chosen for the BB (MCNP universe u = 882). All the remaining geometry definitions were kept unchanged except for the equatorial port (universe u = 210 in the MCNP model) and part of the bioshield and cryostat (u = 900), which were adapted to include the models created for the equatorial port, defined in Section 3.3.

**Figure 5:**
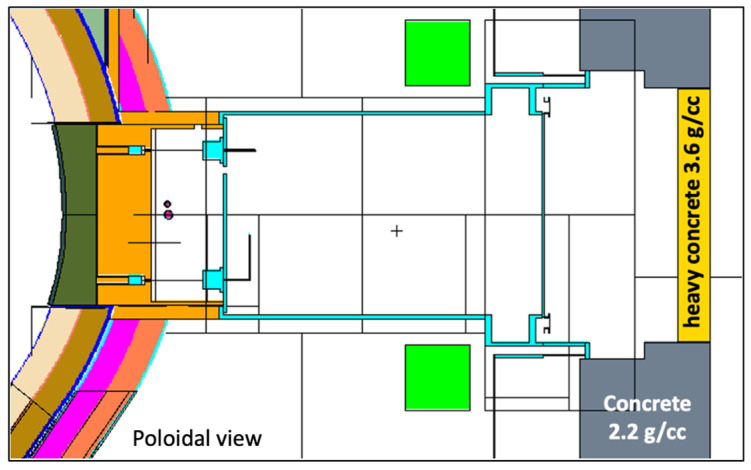
Detail of the MCNP reference model used in the simulations of equatorial port diagnostics, showing the EP and the bioshield plug.

The equatorial port of the reference model is shown with more detail in Figure 5. The bioshield is made of concrete and has a thickness of 2 m, while the bioshield plug is a 50 cm thick slab of heavy concrete with a density of 3.6 g/cm^3^. As there were no CAD models available with details of the DEMO bioshield plugs at the start of this work, the design available in the reference neutronics model was used, with the addition of the diagnostic duct openings in the bioshield plug.

### 2.3. Weight Window Generation

**Figure 6:**
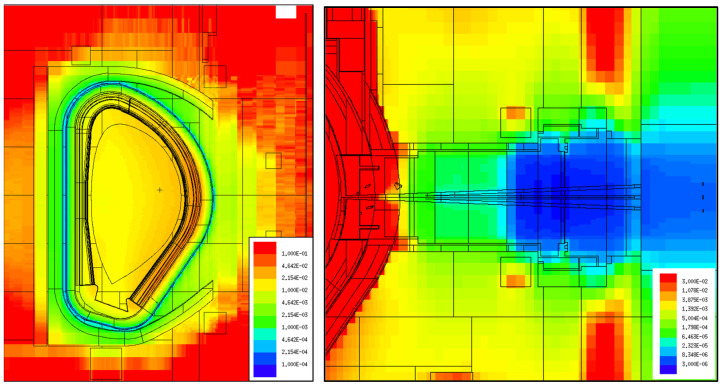
Weight windows used in the simulations. **Left**: Faraday sensors, installed on the outer layer of the VV. **Right**: EP diagnostics.

Common to all cases studied in this paper was the use of weight windows for variance reduction, to reduce the statistical errors of the simulation results. This is a crucial point when the aim is to calculate fluxes or loads at large distances from the plasma, as is the case with most simulations presented here. Two examples are provided in Figure 6. On the left, the weight windows were tuned to calculate the fluxes and loads in the Faraday sensors, using the reference model of Figure 3. In this case, the weight window generator of MCNP was used, after multiplying the density of all materials in the model by 1/10. In subsequent simulations, the material densities were progressively increased (1/5, 1/2 and finally 1), optimizing the weight windows at each step. This allowed us to bias the simulations towards the outer surface of the VV (from red to blue), where the sensors are located. For gammas, this weight window mesh was duplicated with the iWW-GVR [46] code and multiplied by 0.1.

Another example is provided on the right side of Figure 6, for equatorial port diagnostics (reference model of Figure 4). In this case the aim was to obtain fluxes in the port cell, more than 12 m away from the plasma. The weight windows were generated with the ADVANTG code [47] and further manipulated with the iWW-GVR tool. They were tuned, in each simulation, to bias the propagation of neutrons and gammas towards the bioshield and the mirrors in the port cell.

## 3. Results

### 3.1. Inner-Vessel Diagnostics

The main objective of this analysis was to estimate the heat loads and the dose rates in the positions where magnetics sensors are expected to be placed, within the inner surface of the VV. Such an estimation, although preliminary, is important to assess the requirements for magnetics sensors. Since one possibility for the integration of magnetics sensors would be to attach them to the back of a blanket or even to a DSC, a comparison is made in this section between results obtained on the back of the blanket and on the back of a DSC. This comparison allows us to simultaneously assess the effect that the introduction of the DSC, the proposed integration approach for reflectometry and ECE in DEMO, would have on the fluxes and loads in the VV. What follows is a first study of neutron fluxes, nuclear heat loads, dose rates and dpa around the plasma with focus on the 60 poloidal locations where magnetics sensors are expected to be installed.

#### 3.1.1. MCNP Models

**Figure 7:**
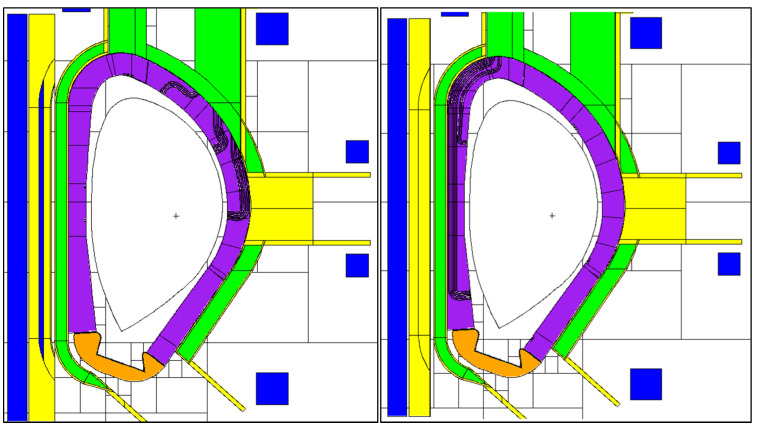
MCNP model of the DSC, containing the antennas and WGs designed for reflectometry. **Left**: Plane y = 10.5 cm. **Right**: Plane y = 6 cm.

**Figure 8:**
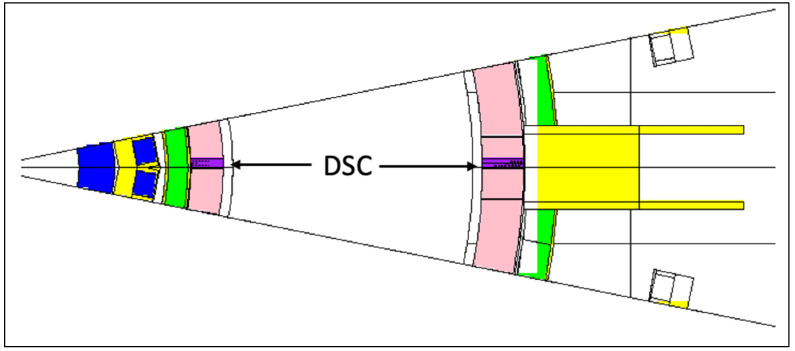
MCNP model of the DSC (plane z = 0).

The model of the DSC is presented in Figure 7 and Figure 8, integrated in the reference model of Figure 2. As described in more detail in [16], the DSC has a thickness of 25 cm in the toroidal direction and the same poloidal shape as the blankets, ~12 m in height and approximately 52 tons in weight (similar density to the WCLL blankets). A homogenized mixture of EUROFER and water was assumed for the DSC, with volume fractions of 83.6% and 16.4%, respectively, representative of a small module with a cooling system studied in a previous work [24]. This water volume fraction is similar to the one in the blanket (15.9%). The MW antennas and waveguides were kept in the geometry, to provide a more conservative estimation and because they have been shown to have a small impact on the neutron fluxes and nuclear loads in the VV.

**Figure 9:**
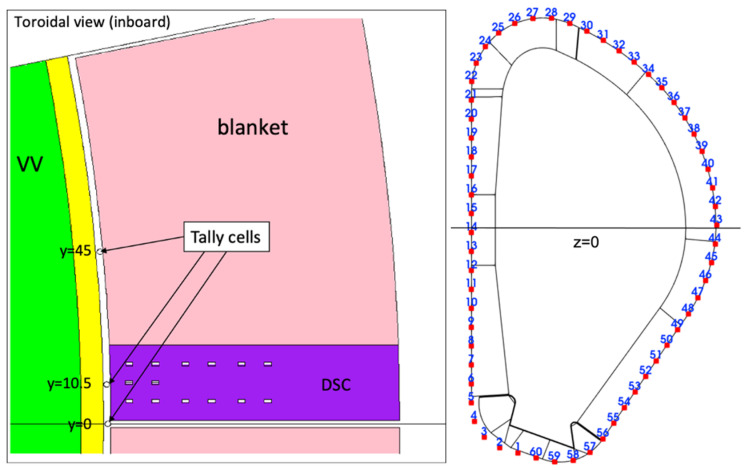
**Left**: Detail of the DSC, highlighting the cells used to tally fluxes, heat loads and dose rates (plane z = 0). **Right**: The 60 poloidal positions around the plasma foreseen for magnetics sensors.

For the first simulations, three tally cells (spheres with 1.5 cm diameters) were created at three toroidal positions (y = 0, y = 10.5 cm and y = 45 cm) in plane z = 0, as illustrated on the left side of Figure 9. These three positions correspond to the point between blanket modules, the middle of the DSC and the middle of the blanket, and were used to evaluate how the distance to the 2 cm gap between blanket segments affects the neutron fluxes and nuclear loads in the magnetics sensors. Afterwards, the fluxes, heat loads, dose rates and dpa in the blankets and DSC were estimated using FMESH tallies with multiplication factors. Using Paraview, the values for each of these quantities were obtained at the 60 poloidal positions where the magnetics sensors are expected to be placed, for the two studied configurations: with the DSC (sensors located behind the DSC) and without the DSC but considering a BB with the full (toroidal) width and having the sensors in the same positions (sensors located behind the blanket). These positions are illustrated on the right side of Figure 9.

The heat loads and dose rates were estimated for the following candidate materials:

- Alumina (Al_2_O_3_)—3.95 g cm^−3^;
- DuPont-951 ceramic (43%at. Al, 31%at. Si, 20%at. O, 6%at. Ca)—3.1 g cm^−3^;
- Aluminum nitride (AlN)—3.26 g cm^−3^;
- Magnesia (MgO)—3.58 g cm^−3^;
- Silicon dioxide (SiO_2_)—2.65 g cm^−3^;
- Silicon nitride (Si_3_N_4_)—3.17 g cm^−3^.

#### 3.1.2. Fluxes, Nuclear Heat Loads and Dose Rates in the Tally Cells

The neutron fluxes, gamma fluxes, nuclear heat loads and dose rates at the three toroidal locations presented in Figure 9 (left) are summarized in Table 1, for the two studied cases (with and without DSC, in which case the DSC portion in Figure 9 is replaced by the blanket). As expected, the neutron and gamma fluxes at the center of the 2 cm gap between BB modules are three to four times higher than the fluxes behind the DSC or the blanket (at y = 10.5 cm). In the middle of the blanket (y = 45 cm), the neutron fluxes are 65% (with DSC) to 74% (without DSC) lower than at y = 10.5 cm. Without the DSC, the gamma fluxes at y = 10.5 cm are a factor of 2.6 higher than at y = 45 cm, due to the increased production of gammas by uncollided neutrons at y = 0 (between blankets). With the DSC, this factor increases to 6.4, due to the increased production of gammas in the DSC. Overall, between the three locations and the two configurations, the neutron fluxes vary between 7 × 10^11^ and 5 × 10^12^ n cm^−2^ s^−1^ and the gamma fluxes vary between 7 × 10^10^ and 1.4 × 10^11^ γ cm^−2^ s^−1^.

Comparing the two configurations, the heat loads and dose rates are very similar when the sensors are located between the blanket modules, as neutrons are the main contributors to the total heat loads and dose rates in that position and the neutron fluxes are similar between the two cases. Behind the DSC/BB (y = 10.5 cm) the heat loads and dose rates are 47% to 69% higher in the DSC configuration, depending on the material. In the position behind the middle of the BBs (y = 45 cm) the heat loads and dose rates are 5% to 9% higher in the case without DSC.

Comparing the three toroidal positions, the loads are much higher between blankets (y = 0) and much lower behind the middle of the blanket (y = 45 cm). Between the blankets the heat loads vary between 60 and 90 mW cm^−3^, while behind the DSC/blanket (at y = 10.5 cm) they vary between 5 and 12 mW cm^−3^. At y = 45 cm the heat loads vary from 2 to 4 mW cm^−3^. Similarly, the dose rates vary between 20 and 30 Gy s^−1^ between blankets, 2 and 3 Gy s^−1^ behind the DSC/blanket (y = 10.5 cm) and 0.7 and 1 Gy s^−1^ at y = 45 cm.

As for the heat loads between different materials, DP-951 and SiO_2_ are the ones with the lower values in general, although the differences are less pronounced behind the blankets. The maximum deviation between the values obtained with each material is ~30%, which is expected given the similar densities of the materials and the comparable atomic weights of their constituent elements. The contribution by neutrons to the loads and dose rates is dominant between the blankets (y = 0), while at y = 45 cm the contributions from neutrons and gammas are similar. At y = 10.5 cm, gammas play the most important role, especially in the case of the DSC, in which they contribute more than 80% of the total heat loads.

**Figure 10:**
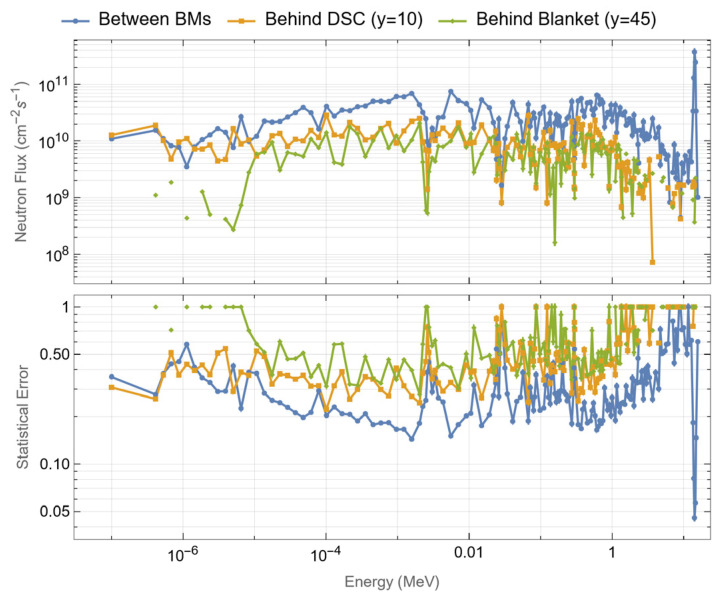
Neutron flux spectra (n cm^−2^ s^−1^) and statistical error fraction behind the DSC and WCLL blanket.

To finalize the comparison, the neutron flux spectra for the DSC case are presented in Figure 10. Although the statistical errors are large in most bins, and therefore unsuitable to be used as input in inventory or activation analyses, the ones related to the total flux are 1.8%, 3.6% and 4.7%, for the cases between blankets, behind the DSC and behind the blanket, respectively. The 14 MeV peak of uncollided neutrons is evident for the tally cell between the blankets (y = 0), vanishing completely in the remaining positions. For the case without the DSC the spectra are very similar and were not included in the plot.

#### 3.1.3. Neutron and Gamma Fluxes in the Sensors

**Figure 11:**
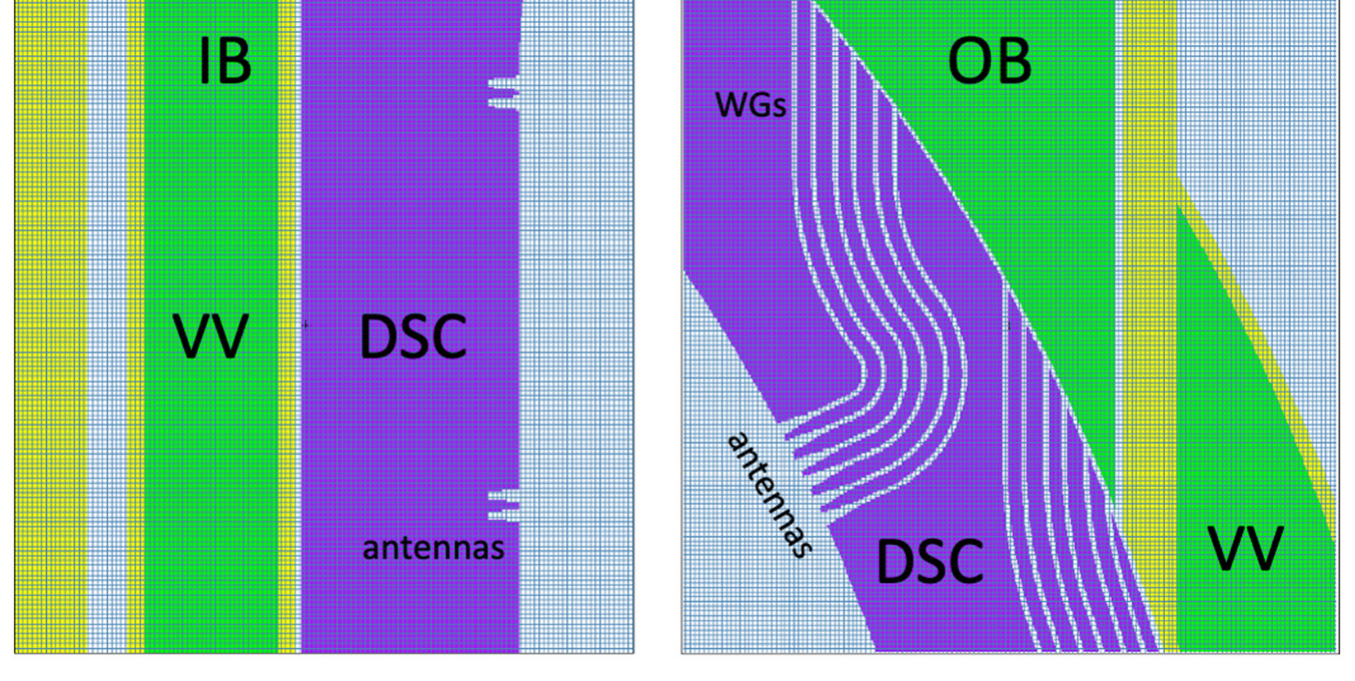
Detail of the meshes used in the simulations.

To assess the variation of the previous quantities with the poloidal location, FMESH tallies were defined for a thin slice between y = 10 cm and y = 11 cm. As shown in Figure 11, the mesh elements are very small (1.7 cm × 1 cm × 1.7 cm), to provide an accurate estimate of the fluxes and loads at each of the 60 positions described in Figure 9. Nevertheless, small deviations between the results presented in Table 1 and the ones obtained with the FMESH tallies are always to be expected, as the elements of the FMESH tallies have a different volume compared to the F4 tally cells. To keep the statistical errors as low as possible with such small elements, 5 × 10^10^ particles were run in each simulation.

**Figure 12:**
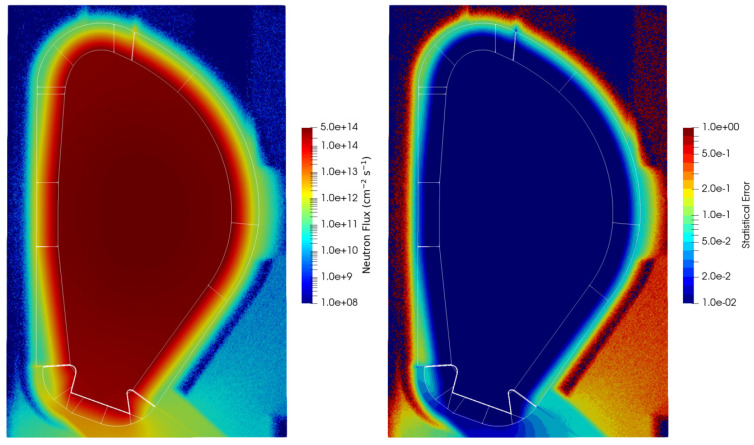
Neutron fluxes (n cm^−2^ s^−1^) and statistical errors behind the WCLL blanket in plane y = 10.5 cm.

The neutron fluxes and statistical errors obtained for the configuration without the DSC are presented in Figure 12. As expected, the fluxes in the first wall are of the order of 3–4 × 10^14^ n cm^−2^s^−1^, while behind the blanket they vary between ~1 × 10^11^ and 1 × 10^12^ n cm^−2^ s^−1^, depending on the position. In general, the statistical errors behind the DSC are less than 10%.

**Figure 13:**
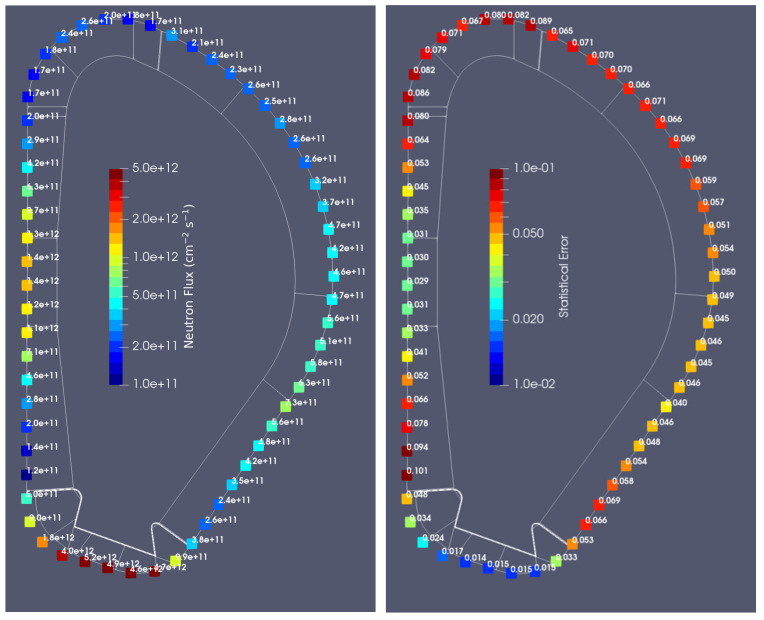
Neutron fluxes (n cm^−2^ s^−1^) and statistical errors behind the WCLL blanket at 60 poloidal locations in plane y = 10.5 cm.

Figure 13 shows the values of the neutron fluxes at the 60 poloidal positions foreseen for magnetics sensors. Values vary from $1.1\times 10^{11}$ n cm^−2^ s^−1^ in position 6 (see Figure 9) to 5.2 × 10^12^ n cm^−2^ s^−1^ below the divertor (more than one order of magnitude of variation). In the equatorial plane, at position 14 (z = −13.5 cm), the neutron flux is 1.4 × 10^12^ n cm^−2^ s^−1^, which agrees with the value provided in Table 1. The largest statistical error is 10.1%.

**Figure 14:**
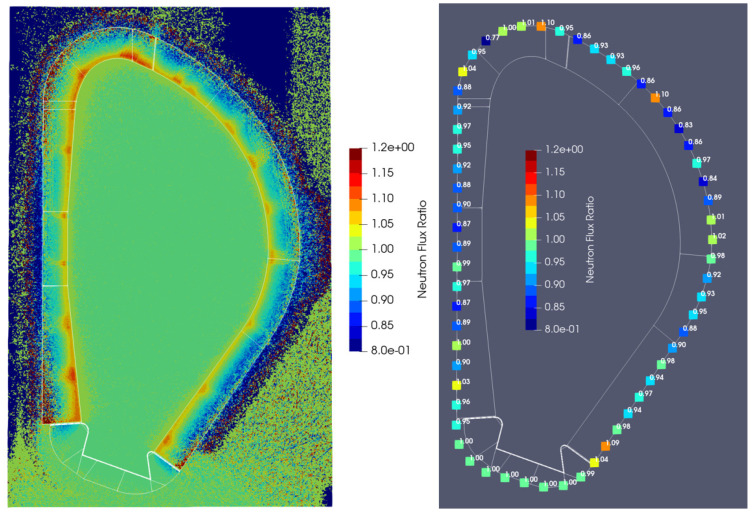
**Left**: Neutron flux ratios (with DSC/without DSC) in plane y = 10.5 cm. **Right**: Values at 60 poloidal locations.

For comparison between the two cases, the ratios between the results obtained with the DSC and without the DSC are presented in Figure 14. The neutron fluxes with the DSC are slightly higher close to the first wall, but lower at almost all positions behind the DSC/blanket. The main conclusion is that the neutron fluxes are very close between configurations, with differences below 20% at most positions.

**Figure 15:**
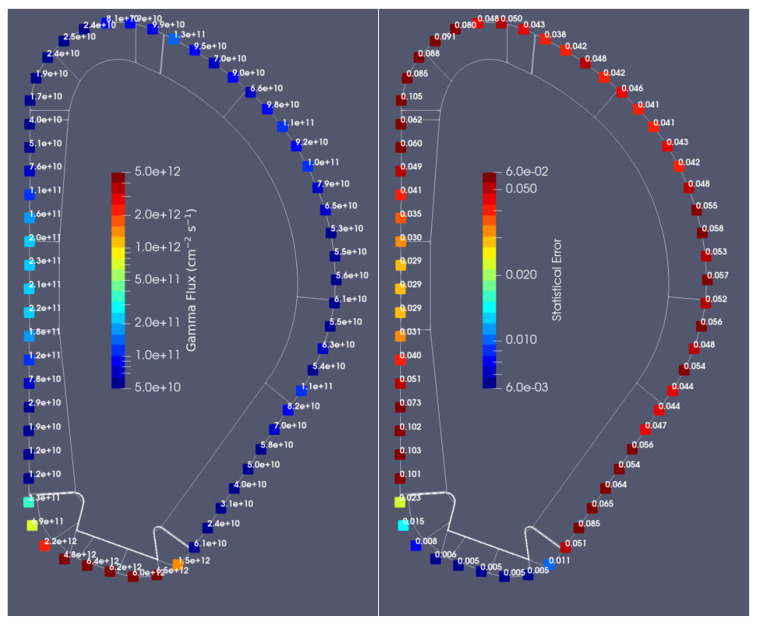
Gamma fluxes (**γ** cm^−2^ s^−1^) and statistical errors behind the WCLL blanket at 60 poloidal locations.

The gamma fluxes behind the blanket are plotted Figure 15. They reach 2 × 10^11^ **γ** cm^−2^ s^−1^ at the equatorial plane (three orders of magnitude lower than at the first wall), while in the divertor region they reach 7 × 10^12^ **γ** cm^−2^ s^−1^. These simulations were run with larger mesh elements (5 cm × 1 cm × 5 cm), to reduce the statistical errors of the simulations, which exceeded 10% in some positions with the initial mesh. Similar values were obtained between the two simulations (smaller and larger mesh elements), with statistical errors ≤ 10% for the larger elements.

**Figure 16:**
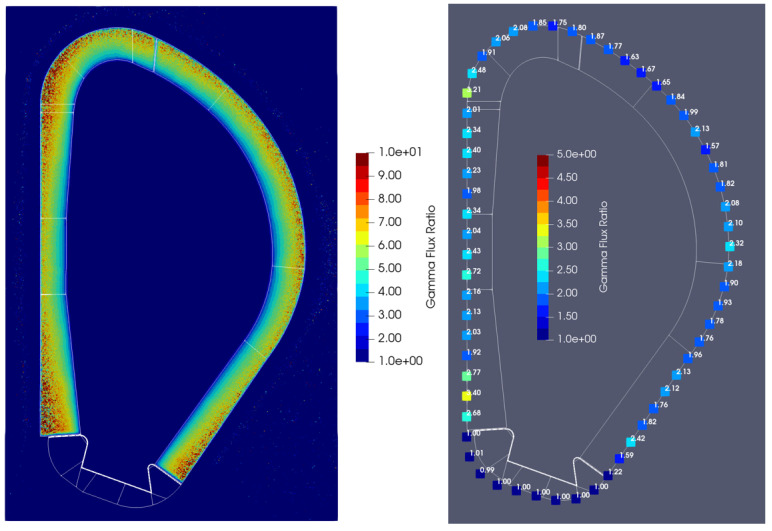
**Left**: Gamma flux ratios (with DSC/without DSC) in plane y = 10.5 cm. **Right**: Values at 60 poloidal locations.

The ratios between the gamma fluxes with and without the DSC are presented in Figure 16. Except for the divertor region—where the ratios are 1—the gamma fluxes increase with the DSC by a factor of 2–3. This is because more gammas are produced in the DSC than in the BB, due to the higher radiative capture cross-sections of iron and chromium (the main constituents of EUROFER) when compared to the radiative capture cross-section of lead (from the WCLL BB).

Since the results of the next sections (nuclear heat loads, dose rates and dpa) were obtained as neutron and gamma fluxes multiplied by conversion factors, the statistical uncertainties presented up to now were considered acceptable and are omitted in the remaining results.

#### 3.1.4. Displacements per Atom in the Vacuum Vessel

**Figure 17:**
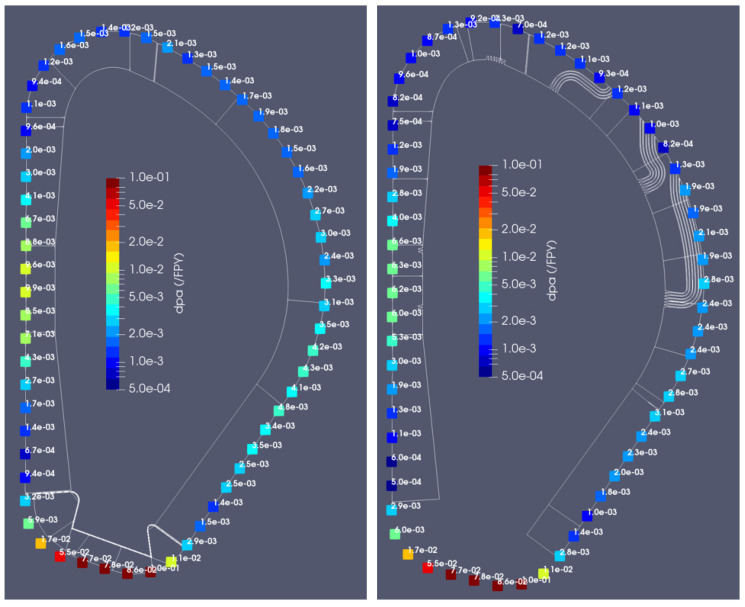
**Left**: dpa/FPY behind the WCLL blanket. **Right**: dpa/FPY behind the DSC.

The dpa values in SS-316 (inner-vessel surface) were also calculated for the two configurations, as shown in Figure 17. The results are normalized per full power year (FPY), with DEMO scheduled to operate over 20 calendar years at an average availability of 30%, which results in a plant lifetime of 6 FPY (1.57 FPY in the first operation phase and 4.43 FPY in the second operation phase) [1]. The dpa values are very small behind either the blanket or the DSC, below 0.01 dpa/FPY in any position (except for the divertor region, where values are much larger). When both configurations are compared, the dpa values are smaller with the DSC, up to a factor of 2. This result indicates once again that the introduction of the DSC, with the current design, does not compromise the integrity of the VV.

#### 3.1.5. Nuclear Heat Loads and Dose Rates

The final step of this analysis consisted in the estimation of the nuclear heat loads and dose rates in the six candidate materials foreseen for magnetics sensors. The nuclear heat loads for the two configurations are shown in Table 2.

As seen before, DP-951 and SiO_2_ are the materials with lower values in general, although the variations between materials are mostly within 40%. As an example, the heat loads at the equatorial plane inboard in the configuration without the DSC vary from 5.3 mW cm^−3^ in SiO_2_ to 7.1 mW cm^−3^ in Al_2_O_3_ (a variation of ~30%). Apart from the divertor region, the highest heat loads are at the equatorial plane inboard (a factor ~3 higher than at the outboard).

On average, excluding the points in the divertor region, the heat loads in the configuration with the DSC are 50% higher than the ones in the configuration without DSC. This is mostly due to the increased gamma fluxes coming from the DSC. The dose rates, obtained by dividing the nuclear heat loads (in mW cm^−3^) by the material density, are very similar between materials, as shown in Table 3.

The results presented in this section show that the DSC leads to an increase in the gamma fluxes and heat loads in the VV without compromising its integrity. The heat loads in the VV obtained with the DSC are well below the limit of 0.3 W/cm^3^, while the dpa values, smaller than 0.01 dpa/FPY at any position, are lower than the ones obtained with the WCLL blanket.

For the magnetics sensors, the increase in the heat loads and dose rates by approximately 50% may have an impact on the integrity of the sensors. The absolute values of the loads and dose rates need to be evaluated by magnetics diagnostic development teams.

#### 3.1.6. Neutron Fluence in Magnetics Sensors: Comparison with ITER

The neutron fluxes in the magnetics sensors presented in Figure 12 vary between 1.1 × 10^11^ n cm^−2^ s^−1^ and 1.4 × 10^12^ behind the blanket and are up to 5.2 × 10^12^ n cm^−2^ s^−1^ below the divertor. Table 4 presents these fluxes integrated over the DEMO operation phases (1.57 and 4.43 FPY), converted to n m^−2^ for comparison with ITER results. The neutron fluences in the ITER in-vessel magnetics sensors are expected to vary in the range 2.5 × 10^24^–5 × 10^24^ n m^−2^ [48], with the fluences in the cable looms reaching up to 6.25 × 10^24^ n m^−2^ close to the upper port and 2.25 × 10^24^ n m^−2^ in the divertor [49]. The fluences behind the blankets presented in Table 4 for the whole DEMO lifetime are comparable to the ones expected for ITER, even though ITER will operate only for 0.54 FPY. This is due to the excellent shielding performance of the WCLL blanket. In the divertor region, the values obtained for DEMO are higher by a factor of 4, although the comparison here is not straightforward, since in the ITER simulations the sensors are installed inside the divertor cassette.

As mentioned before, with the HCPB blanket the fluences in the sensors would be increased up to a factor of 10 or more, which would increase the loads when compared to ITER. Furthermore, the WCLL blanket design changes every year, and a reduction of the blanket dimensions cannot be ruled out at this stage. It is also important to highlight that the present analysis is very preliminary, as it assumes homogeneous material compositions in the blanket and DSC. Therefore, it is important to repeat the analysis when the new models are available, if possible using a fully heterogeneous model of the WCLL blanket [50], with the required adaptations to provide space for the inclusion of magnetic sensors.

### 3.2. Ex-Vessel Diagnostics (Faraday Sensors)

**Figure 18:**
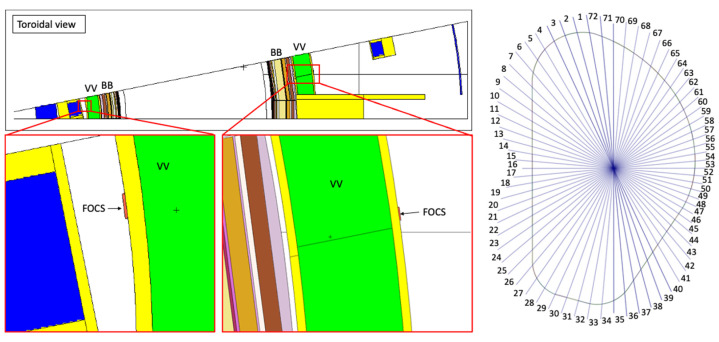
**Left**: Location of the FOCS in the MCNP model. **Right**: Poloidal positions used in the simulations to tally fluxes and energy deposition.

As in ITER [51], the DEMO fiber optics current sensor (FOCS) diagnostic is planned to be installed on the outer surface of the VV, with the aim to provide information on the plasma current during long plasma discharges. To model this diagnostic, a thin layer was added close to the middle section of the VV in the reference model of Figure 3, as illustrated in Figure 18. This layer is a full poloidal segment, with 1 cm in the radial direction and 9.5 cm in the toroidal direction. It was used to calculate neutron and gamma fluxes at different poloidal positions, illustrated on the right side of Figure 18. The layer was first split at the equatorial level, and the remaining planes were obtained by rotating the preceding one by 5 degrees. In this way, 72 cells were added to the MCNP model, after conversion of this CAD model with SuperMC.

The only change made to the reference model was the addition of the 72 cells, which were filled with silica to calculate the heat loads in the optical fibers, with a density of 2.32 g/cm^3^. The remaining modeling options were kept unchanged.

A preliminary simulation was run to determine the volumes and masses of each of the 72 cells, using a voided geometry. The statistical errors of the volumes were kept below 0.5%.

#### Fluxes, Heat Loads and Dose Rates in the Sensors

To obtain statistical errors below 10% in all cells, 4 × 10^10^ particles were simulated, using weight windows. The neutron and gamma fluxes in each cell, along with the nuclear heat loads and dose rates, are presented in Table 5. There are only two cases (gamma flux in position 54 and neutron flux in position 58) in which the statistical error was above 10%. The largest fluxes, heat loads and dose rates were obtained in the cells below the divertor, as expected from the previous simulations for magnetics sensors. Similar flux values were also obtained in a recent divertor study [52]. In position 32, the fluxes reach 1.31 × 10^11^ n/cm^2^/s and 2.60 × 10^10^ γ/cm^2^/s, while the nuclear heat load reaches 0.16 mW/cm^3^ (29% by neutrons and 71% by gammas). The dose rate, calculated by dividing the nuclear heat load obtained with MCNP (1.63 × 10^−4^ W/cm^3^) by the material density (2.32 g/cm^3^) and multiplying it by 1000 (g/kg) × 3600 × 24 × 365.25 (s/FPY), reaches 2.2 MGy/FPY. Considering that the first DEMO operation phase corresponds to 1.57 FPY, and the second phase to 4.43 FPY, in the 6 FPY of DEMO lifetime that section of the FOCS would be exposed to 13.2 MGy (3.5 + 9.7). This value exceeds the 10 MGy considered as a conservative upper limit for the FOCS lifetime dose in ITER [53]. Nevertheless, this happens only in the divertor region, which is not modeled as accurately as the blanket in these simulations, and where the design is not well defined and the shielding has not been optimized. In the remaining regions, the dose rates are lower, by up to almost three orders of magnitude.

**Figure 19:**
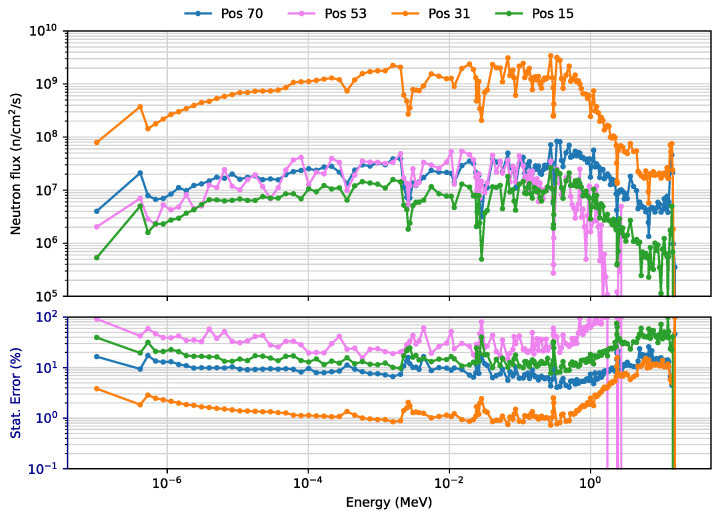
Neutron fluxes (n/cm^2^/s) and statistical errors (%) in 4 FOCS positions (WCLL blanket).

**Figure 20:**
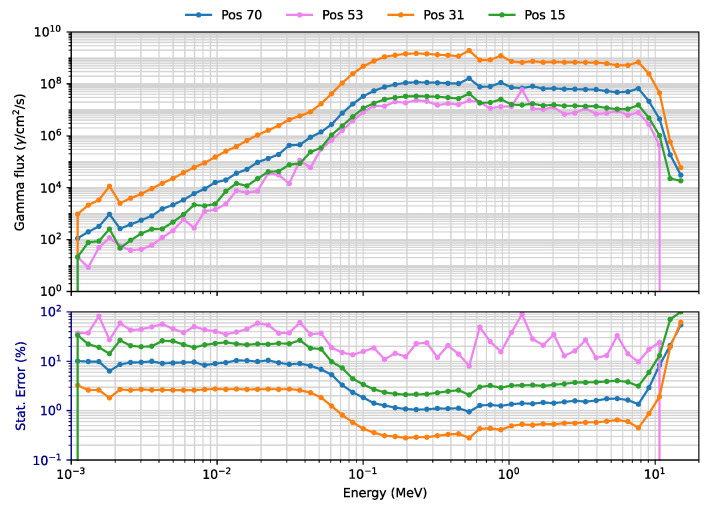
Gamma fluxes (γ/cm^2^/s) and statistical errors (%) in four FOCS positions (WCLL blanket).

This is further illustrated in Figure 19 and Figure 20, which show the neutron and gamma flux spectra in four positions, two at the equatorial port level (15 inboard and 53 outboard), one in the divertor region (31) and the remaining one above the plasma (70). In the divertor region the fluxes are clearly higher when compared to the other positions, for both neutrons and gammas. In position 53 (but also 15 and 31), the statistical errors in the bins are inevitably large, due to the very small binning and the blanket thickness in that area. Nevertheless, the statistical errors in the total neutron and gamma fluxes are only 4.8% and 4.5%, respectively.

The possibility of bringing the FOCS to the inner surface of the VV has also been discussed recently. However, the current results show that the sensors would not be able to withstand the radiation levels, as the neutron fluxes inside the vessel would be around three orders of magnitude higher than ex-vessel [1].

### 3.3. Equatorial Port Diagnostics

The EP configuration studied in this work was based on one of the port integration proposals presented in [54], for an EP housing the following three diagnostics:

- High-resolution core X-ray spectroscopy;
- Near-ultraviolet, visible and infrared divertor monitoring;
- Pellet monitoring.

**Figure 21:**
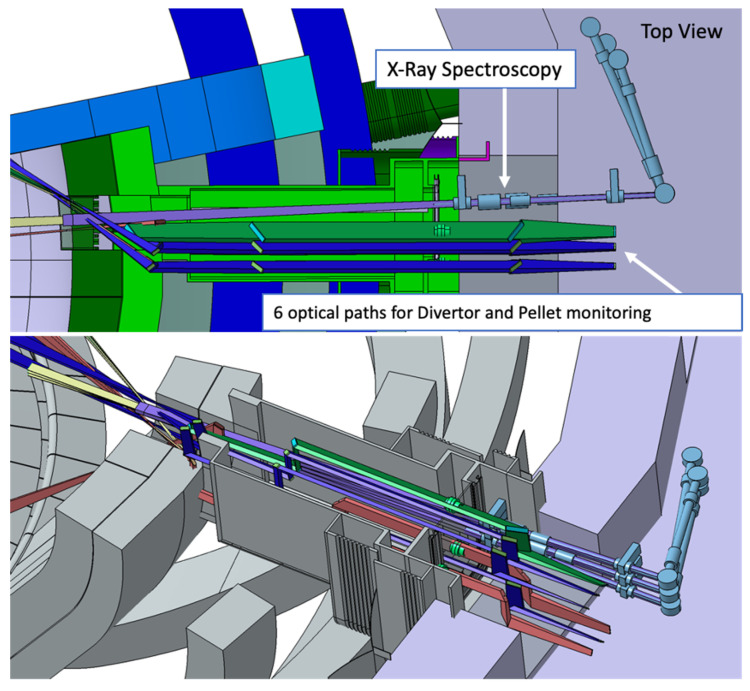
EP configuration with the X-ray spectroscopy (light blue), divertor monitoring (X-point and outer divertor tangential line-of-sight in purple, outer divertor surface views in red) and pellet monitoring (green) systems.

In this integration proposal the six optical paths of the divertor monitoring and pellet monitoring systems are grouped together on the left side of the port (when looking towards the plasma) in two rows with three paths each. The X-ray spectroscope is placed on the right side, with the ducts angled slightly in the EP to increase the space for the port cell optical components of the other systems. This setup is presented in Figure 21.

**Figure 22:**
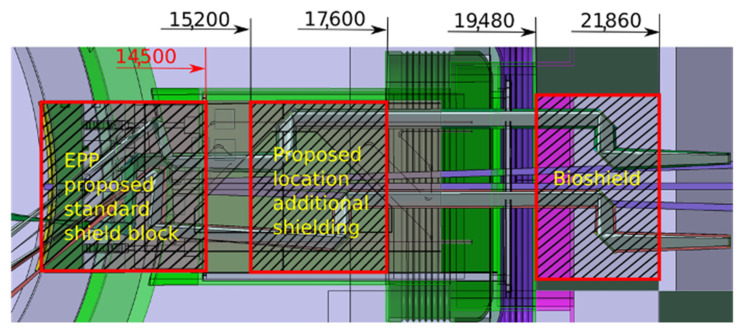
Possible radiation shielding locations proposed for the EP (units in mm).

The objective of this study was to implement this EP configuration in MCNP and evaluate the neutron and gamma fluxes through these diagnostic ducts into the port cell, after the bioshield, testing possible shielding configurations based on the proposals of reference [54], illustrated in Figure 22. These proposals include the standard equatorial port plug shield block (reinforced if needed) and additional shielding in the mirror doglegs along the diagnostic ducts, in the middle of the port and in (or possibly before or after) the bioshield plug. EUROFER and stainless steel were considered for the EPP shield block, while boron carbide (B_4_C) shielding trays similar to the ones foreseen for the EP diagnostics shielding modules (DSMs) of ITER [55] were considered for the middle of the port, due to their shielding efficiency and lower weight.

The ducts from the diagnostics presented in Figure 21 were simplified in ANSYS SpaceClaim, through the removal of details from the vacuum windows and vacuum extensions (only the ducts and the mirrors were left in the model) and of all the spline surfaces present in the model. The design of the EP components was adapted from reference [56], which has the same shape and is compatible with the MCNP reference model.

**Figure 23:**
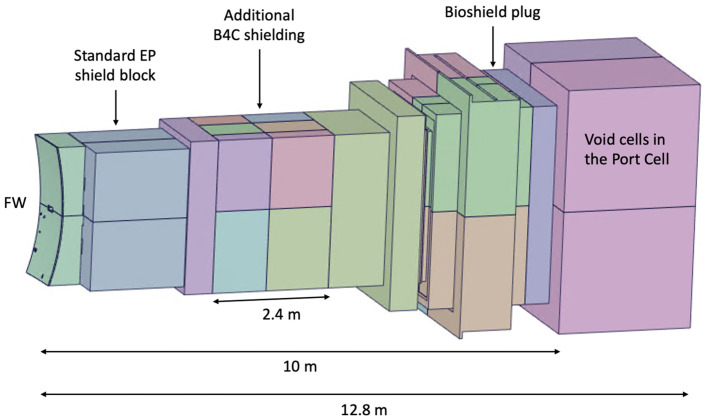
CAD model of the EP used in the simulations.

**Figure 24:**
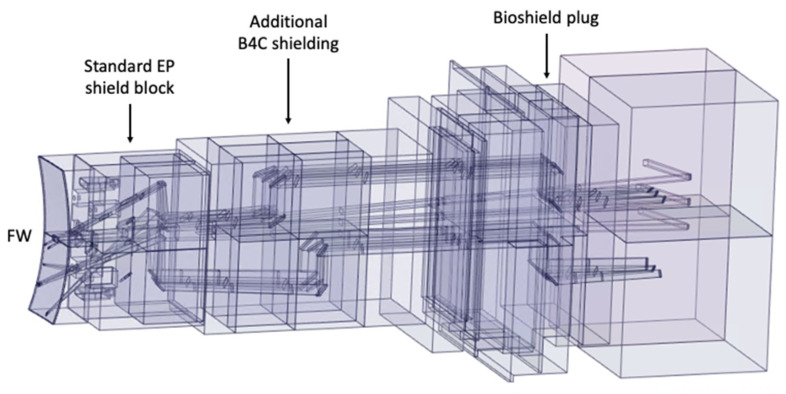
CAD model of the EP used in the simulations with transparent cells, showing the diagnostic ducts.

All components of this model were filled, and the EP diagnostic ducts were carved inside. The result is shown in Figure 23. Some of the cells of Figure 23 were dimensioned to be filled with shielding in the MCNP model—a thickness of 2.4 m was reserved in the middle of the port for the B_4_C shielding—while others were designed to be void cells. The complexity of the ducts in the neutronics CAD model is illustrated in Figure 24, where the cells are represented with transparency. One of the main challenges of this work was the generation of this CAD model, free from splines and small surfaces, ready to be converted to MCNP.

**Figure 25:**
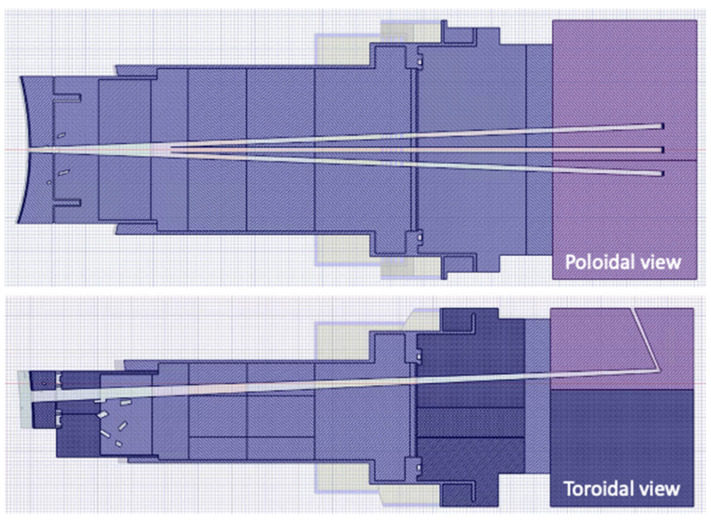
X-ray spectroscopy ducts along the EP.

The diagnostic with the largest openings is the X-ray spectroscopy system, with a first wall opening of 23 cm × 10 cm (230 cm^2^) that spreads into three ducts behind the first wall. As illustrated in Figure 25, these ducts are straight paths from the plasma to the port cell, with openings in the bioshield plug of 10.6 cm × 10 cm (106 cm^2^). As there are no doglegs in this diagnostic, direct neutron streaming is expected through these ducts.

The remaining diagnostics have much smaller openings in the first wall (all below 28 cm^2^). Furthermore, they have doglegs, which will reduce streaming to the port cells, as shown before [25].

**Figure 26:**
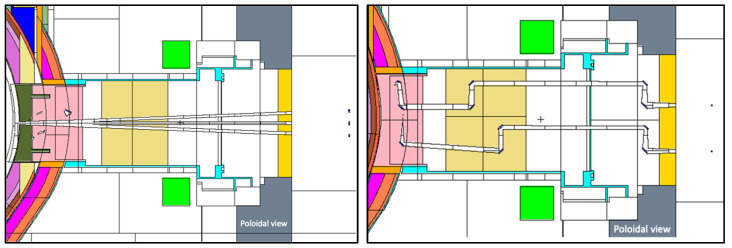
MCNP model of the EP. **Left**: X-ray spectroscopy ducts. **Right**: Near-ultraviolet (bottom) and visible (top) divertor spectroscopy ducts (poloidal view, plane y = −15 cm).

The converted model of the port was integrated in the reference model of Figure 4. The result is presented in Figure 26, for a plane in the middle of the X-ray spectroscopy ducts (left) and for plane y = −15 cm (with the near-ultraviolet and visible divertor spectroscopy ducts). It also illustrates the reasoning behind the shielding distribution inside the port: a first block was added to the standard shielding of the EPP (pink), which contains the first dogleg for all the diagnostics except the X-ray spectroscopy system, and a second block (of B_4_C) was added in the middle of the port (yellow), to shield the second dogleg. Due to the low thickness of the bioshield plug, the third dogleg is not shielded in the studied configurations, although an additional shielding layer could be envisaged for this dogleg, placed in front of the bioshield plug.

**Figure 27:**
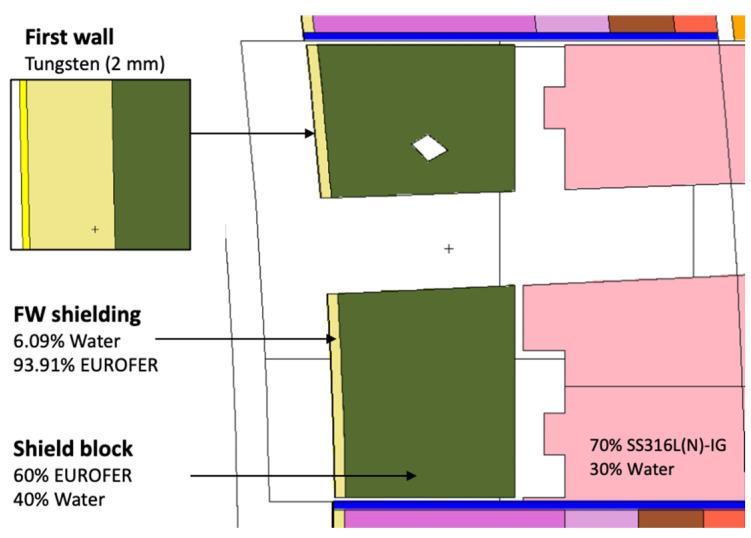
Materials used in the MCNP model of the equatorial port.

Most of the materials used in the equatorial port model are summarized in Figure 27. The first wall has 2 mm thick armor made of tungsten, with a second layer of 6.09% water and 93.91% EUROFER, taken directly from the definition of the WCLL BB which follows the material distribution set out in [57]. The definition for the first wall shield block behind it was adopted from the technical specification for the equatorial outboard limiter [58]: 60% EUROFER and 40% water. For the remaining shielding behind this block a mixture of 70% SS316L(N)-IG stainless steel and 30% water was assumed, while in the second shielding block (yellow in Figure 26, second dogleg) a homogenized mixture of 65% B_4_C, 10% stainless steel and 25% void (to account for the spacings between the components) was defined, with an effective density of 2.28 g/cm^3^ [55]. This mixture represents the B_4_C shielding trays used in the ITER DSMs for the equatorial ports [55].

The mirrors were set to EUROFER, while the remaining components were kept with the same materials used in the equatorial port components of the MCNP reference model.

#### 3.3.1. Neutron Fluxes, Gamma Fluxes and Dose Rates in the Port Cell

**Figure 28:**
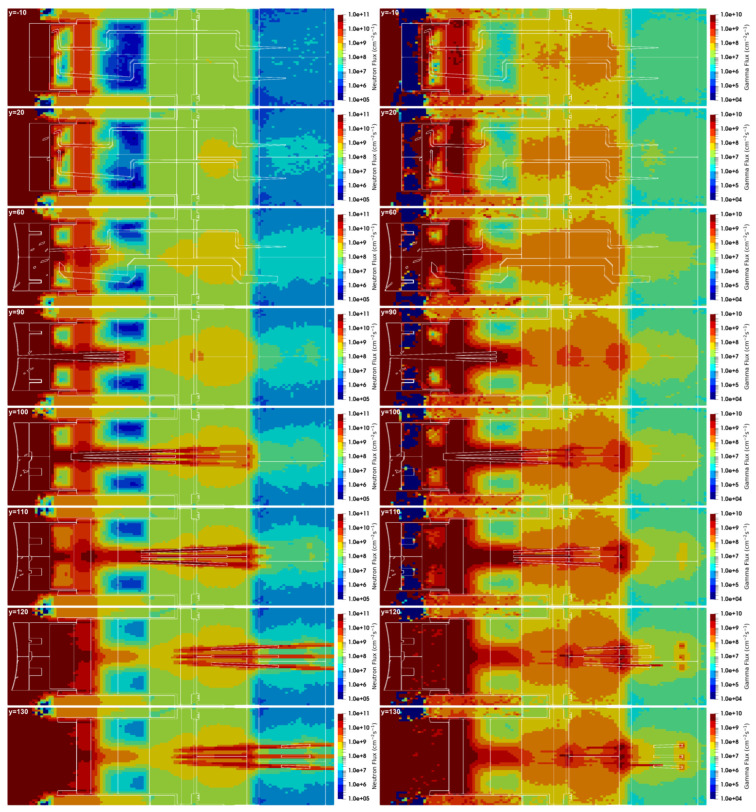
Neutron and gamma fluxes (cm^−2^ s^−1^) in plane y for the configuration with diagnostics in the EP.

**Figure 29:**
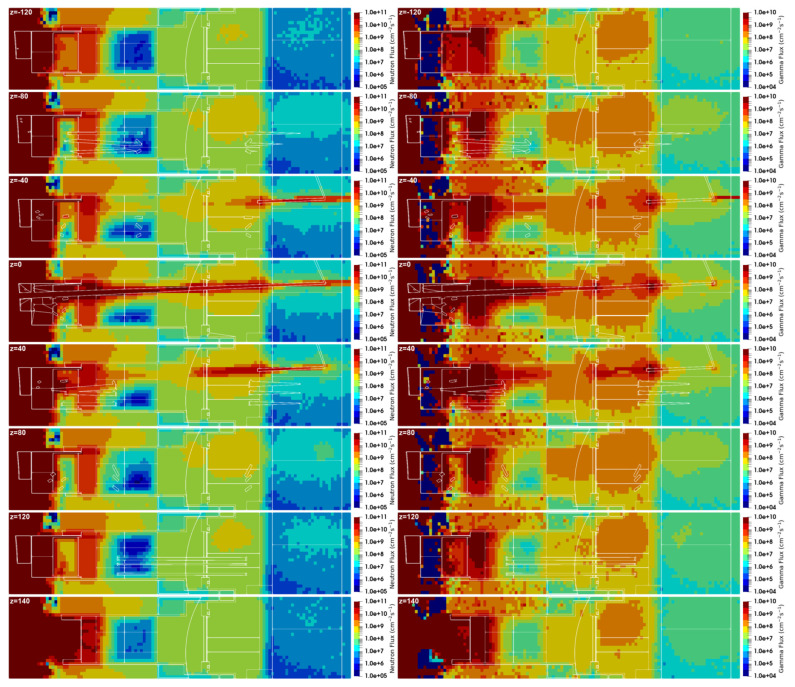
Neutron and gamma fluxes (cm^−2^ s^−1^) in plane z for the configuration with diagnostics in the EP.

**Figure 30:**
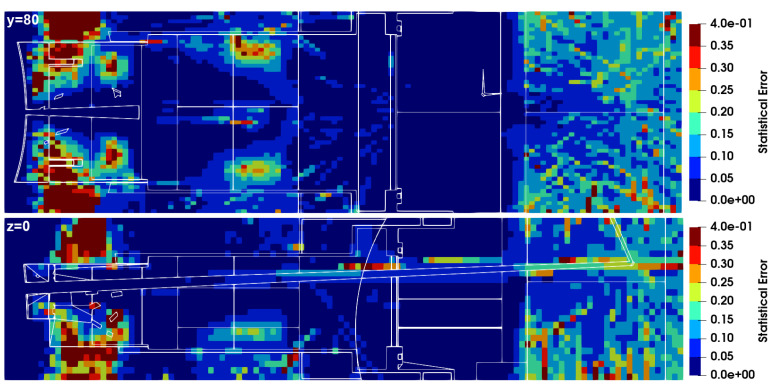
Statistical errors of the neutron fluxes, for the planes y = 80 cm and z = 0.

The neutron and gamma fluxes for this equatorial port configuration are presented in Figure 28 and Figure 29 for several planes y and z, with the neutron flux statistical errors for the planes y = 80 cm and z = 0 presented in Figure 30. As expected, there is substantial neutron streaming through the X-ray spectroscopy ducts, reaching the port cell through the straight paths. This is visible mostly around planes y = 120 cm and z = 0. The neutron fluxes reaching the inner surface of the bioshield through these ducts (the one in z = 0 is used for this estimation) are of the order of 2 × 10^10^ n cm^−2^ s^−1^, decreasing to 4 × 10^9^ n cm^−2^ s^−1^ in the mirror behind the bioshield. The gamma fluxes reach 3 × 10^9^ γ cm^−2^ s^−1^ at the inner surface of the bioshield and 2 × 10^9^ γ cm^−2^ s^−1^ and 2 × 10^8^ γ cm^−2^ s^−1^ in the mirror, while the dose rates in silicon obtained in these positions were 2E6 Gy/FPY and 5E5 Gy/FPY, respectively. The flux and dose rate values in the port cell are more than three orders of magnitude higher than those obtained with the reference model of the port without diagnostics (neutron fluxes below 1 × 10^7^ n cm^−2^ s^−1^ were obtained in the port cell with the reference model). For the remaining port diagnostics, the design of the first doglegs is effective to reduce the streaming, as shown in a previous study [25].

The statistical errors of the neutron fluxes, presented in Figure 30, are below 10% in most of the regions of interest. As expected, they increase along the ducts, even though the weight windows were fine-tuned to increase statistics in the port cell. Due to the distance of more than 12 m between the plasma and the port cell mirrors, it was not possible to have statistical errors below 10% in all the regions of the studied configurations. Nevertheless, F4 tallies were added at the main positions of interest (bioshield and port cell mirrors), and the flux values discussed in the previous paragraph were confirmed, with statistical errors between 3% and 10%.

The shutdown dose rates in the port cells were not calculated, for two reasons: (1) the lack of access, at this stage, to R2S/D1S codes for this kind of calculation in DEMO and (2) the fact that such a calculation would always be far from accurate, as the bioshield plugs and their penetrations, as well as the port cells, have not been designed yet (material activation would require accurate designs of the systems that will populate these rooms). On the other hand, there are no limits defined for the neutron and gamma fluxes in the port cells in the DEMO Nuclear Analysis Handbook [1], which defines a limit of 100 μSv/h in the port cell 12 days after shutdown. However, it can be anticipated, based on experience from ITER, that this limit will be greatly exceeded with the neutron streaming predicted for the X-ray spectroscopy ducts. Another open issue is the radiation limits that the vacuum windows and the optical fibers can withstand, as well as the locations where electronics are required, since in the present design the limits of 100 n cm^−2^ s^−1^ and 10 Gy of cumulative dose could only be enforced with large amounts of shielding in the port cell, or if the electronics are placed far from the streaming paths. In any case, before the other port diagnostics can be studied in more detail it is important to evaluate whether it is possible for the X-ray spectroscopy system to operate with smaller ducts or alternative configurations, to reduce streaming.

The priority was then to understand the effect of the duct cross-section on the neutron and gamma streaming through the port, to provide a guideline for diagnostic design. For this, a sensitivity analysis was carried out for straight ducts from the plasma to the port cell. The results are presented in the next section.

#### 3.3.2. Sensitivity Analysis for Straight Ducts in the Equatorial Port

For this analysis, the equatorial port model presented above was used, with all the diagnostics removed and with only one duct (centered at z = 0 and y = 70 cm). Using that model, neutron and gamma flux spectra were calculated as a function of the duct size, for duct cross sections ranging from diameters of 3 cm up to the size of the X-ray ducts (23 cm toroidal × 10 cm poloidal). For these simulations, weight windows were used in conjunction with source biasing parameters produced by ADVANTG and F5 tallies, i.e., “next event estimators”. Using F5 tallies, each time a source particle is created, or at any collision event, a deterministic estimation is made for the flux contribution at the detector point [59]. This makes F5 tallies ideal for the kind of simulation performed here, with straight ducts from the plasma to the port cell. They slow down the simulations considerably and do not allow the production of mesh tallies but yield accurate results with extremely low statistical errors. Due to the simulation time that F5 tallies require, only two were used in each simulation, for two points after the bioshield plug: at the outer surface of the plug (x = 2200 cm, y = 70 cm, z = 0) and 2 m away from that surface (x = 2400 cm, y = 70 cm, z = 0).

**Figure 31:**
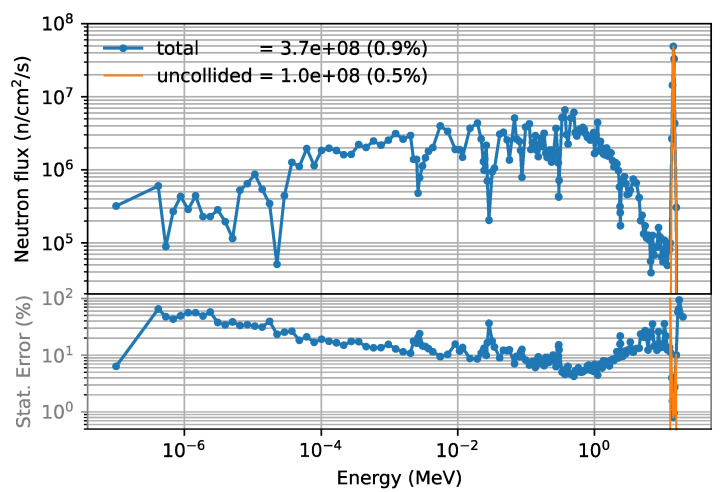
Neutron flux (n/cm^2^/s) and statistical error 2 m behind the bioshield plug (x = 2400 cm, y = 70 cm, z = 0), for a circular duct with r = 1.5 cm.

Figure 31 shows the neutron flux spectrum 2 m behind the bioshield plug for a circular duct with r = 1.5 cm. The total flux value is 3.7 × 10^8^ n cm^−2^ s^−1^, with a very small statistical error of 0.9%. The spectrum also shows that the statistical errors are smaller than 10% for some energy bins, and below 1% at 14 MeV. Also presented is the spectrum of uncollided neutrons—bins around 14 MeV—which corresponds to those neutrons that travel from the plasma to the port cell without any interactions. In these bins, the statistical errors are very small, in some cases below 1%. As will be shown later, the total flux value is compatible with the results obtained in the previous section.

**Figure 32:**
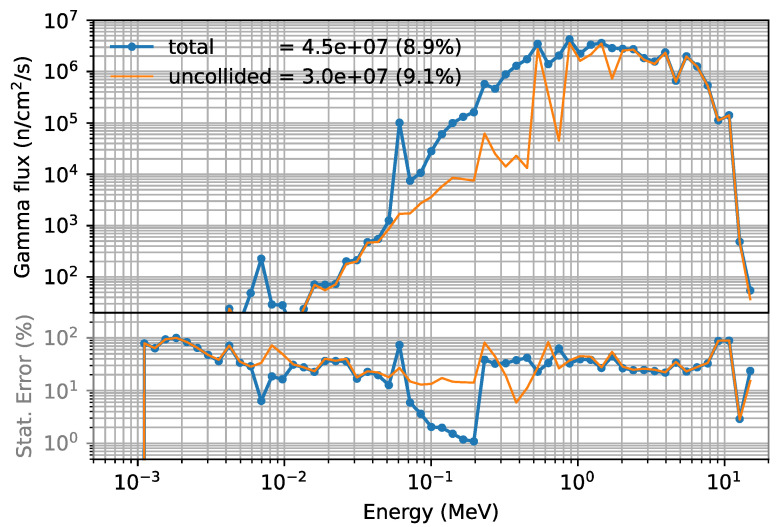
Gamma flux (γ/cm^2^/s) and statistical error 2 m behind the bioshield plug (x = 2400 cm, y = 70 cm, z = 0), for a circular duct with r = 1.5 cm.

The F5 tallies also allowed us to calculate the gamma spectra in the port cell, as shown in Figure 32 for the same case and position as before. The total gamma flux (4.5 × 10^7^ γ cm^−2^ s^−1^) is almost one order of magnitude lower than the neutron flux. The statistical errors are larger in this case, but still below 10%. The uncollided spectrum refers to gammas that were created somewhere in the geometry and traveled to the port cell without interactions. For both cases (neutron and gammas), reducing the statistical errors below 10% in all bins would be mandatory if these results were to be used as input in inventory or activation calculations; however, this would be prohibitive in terms of computational resources. Furthermore, the “collided” part of the spectrum depends heavily on the shielding configurations, which are very preliminary at this stage.

Similar spectra were calculated for several cases: circular ducts with radii between 1.5 cm and 4 cm (0.5 cm increments) and rectangular ducts of 10 cm in the poloidal direction and several toroidal lengths. The height of these ducts (10 cm) was chosen to be the one projected for the X-ray spectroscopy. The toroidal lengths were varied from 0.71 cm to 23 cm (the initial length foreseen for the X-ray ducts). The first five toroidal lengths, up to 5 cm, were selected to match the area of the circular ducts, to evaluate the effect of the duct shape on the fluxes. After 5 cm, four additional lengths were tested: 10 cm, 15 cm, 20 cm and 23 cm.

The results are presented in Table 6 (neutron fluxes) and Table 7 (gamma fluxes). Looking at the bioshield surface, the neutron fluxes vary from 5.38 × 10^8^ n cm^−2^ s^−1^ for a circular opening with r = 1.5 cm to 3.91 × 10^9^ n cm^−2^ s^−1^ for r = 4 cm. Comparing these values with the corresponding areas for rectangular ducts (toroidal lengths up to 5 cm), it becomes clear that the shape of the duct has no effect on the fluxes; very similar values were obtained for the same areas. When the toroidal length of the rectangular duct is increased to 23 cm, the fluxes increase to 1.9 × 10^10^ n cm^−2^ s^−1^ (or 1.2 × 10^10^ n cm^−2^ s^−1^ 2 m away from the bioshield). This is in excellent agreement with the results shown in the previous section, where fluxes of 1.2 × 10^10^ n cm^−2^ s^−1^ were estimated at the mirror location in the center duct of the X-ray diagnostic.

When comparing the total flux with the uncollided flux, a ratio between 3.5 and 4 is found between the two for all cases. While the total flux will be affected by the EP shielding configuration, the uncollided flux will be similar regardless of the EP design.

**Figure 33:**
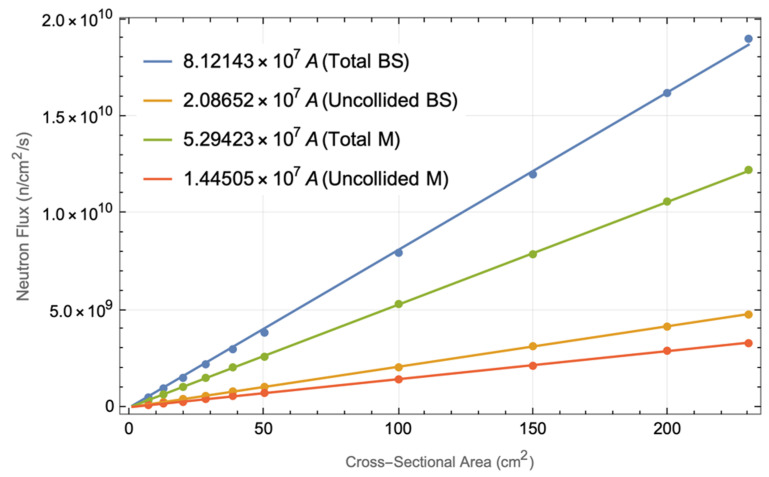
Neutron flux variation with the cross-sectional area, for rectangular ducts.

Figure 33 shows the neutron fluxes plotted against the cross-sectional area of the ducts for the rectangular configuration (the results for the circular ducts are very similar and were omitted). The flux varies linearly with the duct area. The fits were obtained using Mathematica [36] for the simple expression f=c A, where f is the flux, A is the area of the duct and c is a constant, and they can be used as a first approximation to estimate the fluxes in ducts with different areas.

The gamma fluxes are a factor of 5–7 lower than the neutron fluxes. In the previous analysis, 2.3 × 10^9^ γ cm^−2^ s^−1^ was obtained for the central duct of the X-ray system, with a statistical error of 15%, while here the flux is 50% higher: 3.47 × 10^9^ γ cm^−2^ s^−1^ (2.5% statistical error). This variation can be explained by the 15% error in the previous simulations, which points to unreliable results.

#### 3.3.3. Neutron/Gamma Cameras, Radiated Power and Soft X-ray Intensity

Two diagnostics are currently expected to use straight ducts with even smaller cross-sections than the ones simulated in the previous section (r = 1 cm): the neutron and gamma cameras [8,9] and the core radiated power and soft X-ray intensity system [10]. The aim of this section is to provide the neutron and gamma fluxes through the different ducts of these systems.

**Figure 34:**
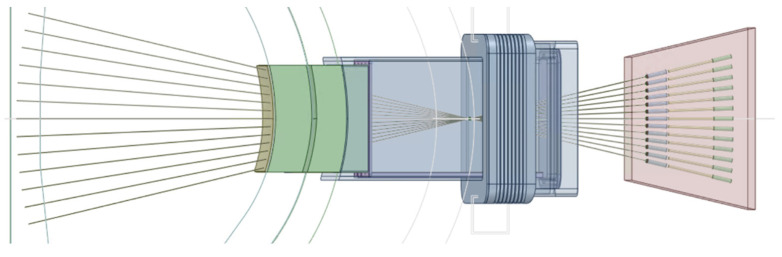
CAD model of the neutron and gamma cameras.

**Figure 35:**
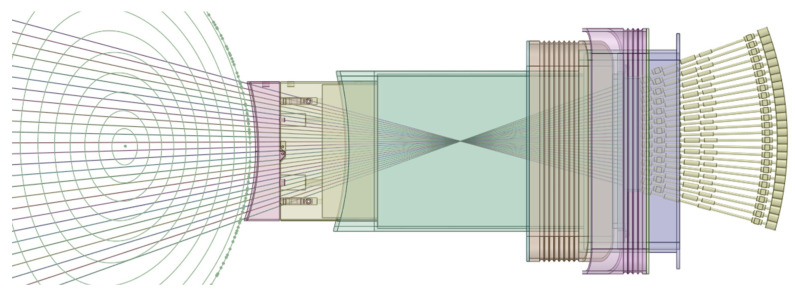
CAD model of the core radiated power and soft X-ray intensity system.

**Figure 36:**
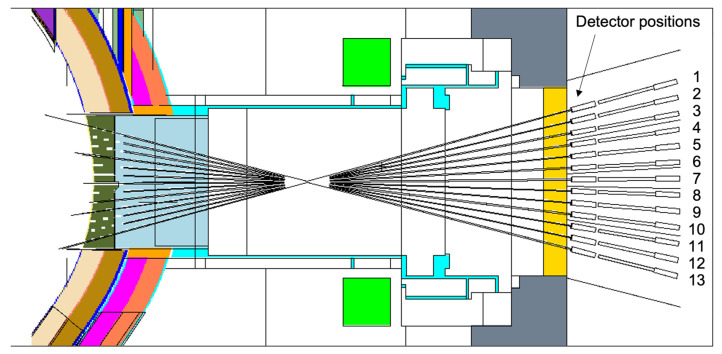
Neutronics model used in the simulations of the neutron/gamma cameras and core radiated power and soft X-ray intensity system (plane y = 50 cm).

The CAD model of the neutron and gamma camera system is represented in Figure 34. It contains 13 ducts with a 1 cm cross-section radius as well as neutron and gamma detectors and a shielding/collimator block enclosing them. It is similar to the CAD model of the core radiated power and soft X-ray intensity system, presented in Figure 35, the main difference being that this updated design of the radiated power system contains 26 ducts instead of 13. The positions of the ducts are also not the same and intersect at different points. Despite these differences, these systems have relatively similar duct configurations, and since the simulations required to estimate the fluxes in the port cells are very CPU-intensive, the configuration presented in Figure 34 (neutron and gamma cameras) was adopted as representative for both systems in this analysis. The MCNP geometry used in the simulations, based on the EP design used in the previous section, is presented in Figure 36.

All the cells within the ducts, including the detectors, were modeled as void, to prevent effects related to neutron or gamma scattering in the detector materials, which are different between the two systems. All materials of the EP were kept unchanged from the reference model.

As before, the simulations were run using the weight windows generated with the ADVANTG code. The source biasing parameters generated by ADVANTG were also added to the reference neutron source. As in the previous section, F5 tallies were used at the 13 detector positions to tally the neutron and gamma fluxes. The heat loads in beryllium, the material proposed for the vacuum window of the radiated power diagnostic, were also calculated with F5 tallies, using conversion factors.

The neutron and gamma fluxes in the 13 positions are summarized in Table 8. As expected, the uncollided neutron flux increases steadily from position 1 (top, 4.2 × 10^7^ n cm^−2^ s^−1^) to positions 6 and 7 (middle, 6.9 × 10^7^ n cm^−2^ s^−1^), decreasing afterwards until position 13 (bottom, 3.9 × 10^7^ n cm^−2^ s^−1^). These fluxes are mostly independent of the EP shielding configuration. The total fluxes are 2.3 to 2.7 times higher than the uncollided fluxes and have a similar trend (except at detector 3, where the flux is slightly higher than in detector 4). The total gamma fluxes are four to five times lower than the total neutron fluxes, except at detector 2, where the flux has an unexpectedly large statistical error. Even though the simulations were performed for 5–7 days with 720 processors in the MARCONI cluster, it was not possible to bring the statistical errors in the gamma fluxes below 10% for all positions. Nevertheless, a trend can be established from the results in the positions where the errors are smaller.

The nuclear heat loads in beryllium are presented in Table 9. As these results were obtained with F5 tallies, the heat loads due to uncollided neutrons and gammas are also presented, along with the total heat loads obtained by summing the neutron and gamma contributions. Since beryllium is a neutron moderator and the neutron fluxes are higher than the gamma fluxes, neutrons have the highest contribution to the total heat loads, exceeding the gamma contribution by more than one order of magnitude. The total heat loads range from 3.0 μW/cm^3^ at position 13 to 5.3 μW/cm^3^ in the central detector positions (6 and 7). The statistical errors are below 1% for all positions.

Due to the computational resources required to run these simulations, it can be anticipated that if more detailed results are required—nuclear heating in different detector volumes, for example—a different strategy should be followed, possibly involving the generation of a secondary source at the exit of the bioshield. The benchmark of such a source could be challenging; however, that work could be simplified by defining sources only at the bioshield openings. Such an approximation seems acceptable at this stage, considering that the contribution of uncollided neutrons (14 MeV) accounts for 86–88% of the neutron heat loads, or 82–83% of the total loads, as estimated with the F5 tallies in MCNP. This approach will be explored in future simulation work.

#### 3.3.4. Alternative Configuration of the X-ray Spectroscopy Diagnostic

As stated in Section 3.3.1, the design proposed for the X-ray spectroscopy system leads to very high neutron and gamma streaming to the port cell. As shown in Figure 28, neutron fluxes up to 2 × 10^10^ n cm^−2^ s^−1^ were predicted to reach the port cell through the large straight ducts (23 cm × 10 cm) of the X-ray spectroscopy system, almost four orders of magnitude higher than in the default EP configuration without diagnostics. These fluxes, along with gamma fluxes one order of magnitude lower, would lead to high dose rates that would exceed the limits in the port cell and that could compromise the integrity of electronic devices in the port cell. The sensitivity study presented in Section 3.3.2 has allowed us to evaluate the effect of reducing the cross-section of the ducts on the neutron and gamma streaming to the port cell. In parallel, alternative diagnostic duct geometries have been investigated, based on flat highly oriented pyrolytic graphite (HOPG) pre-reflectors, as included in the design of a similar system for ITER [60]. Although the feasibility of these alternative configurations is still questionable—due to low reflectivity and the possibility of increased radiation streaming to the magnets [61]—it is worthwhile to evaluate if such configurations would address the radiation streaming issue. The neutronics simulations presented in this section aim to contribute to a better understanding of the different design options for the X-ray spectroscopy system.

**Figure 37:**
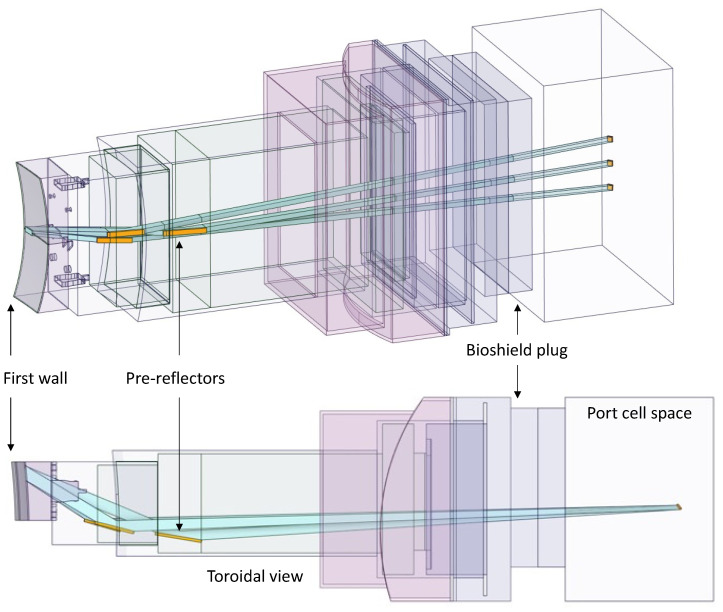
CAD model of the EP used in the simulations with transparent cells, showing the pre-reflectors and the diagnostic ducts of the X-ray spectroscopy system.

The reference model used for the simulations is the same as presented in the previous sections. The neutronics CAD model of the system, including the HOPG mirrors to minimize streaming, is presented in Figure 37. The three ducts maintain the previous dimensions (23 cm × 10 cm), but not in straight paths from the plasma to the port cell, as before. Since the objective was to evaluate the streaming through the X-ray ducts, the other systems, which have very small contributions to the total fluxes in the port cell, were not included in the model.

**Figure 38:**
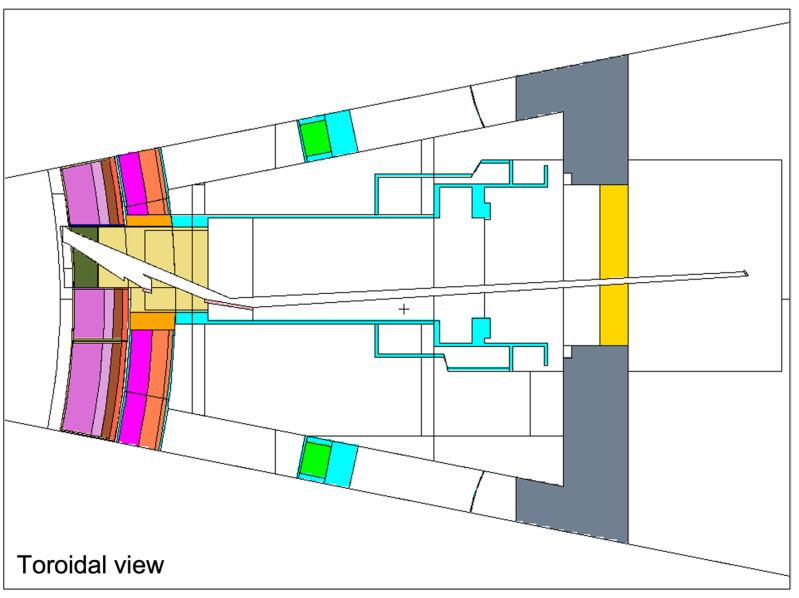
MCNP model of the EP, showing the X-ray spectroscopy ducts (plane z = 1 cm).

The MCNP model is presented in Figure 38, for plane z = 1 cm. The crystal Bragg reflectors in the port cell were also included, and used to tally the neutron and gamma fluxes that cross the bioshield plug. All the simulations were run using weight windows generated with the ADVANTG code and further manipulated with the iWW-GVR tool.

**Figure 39:**
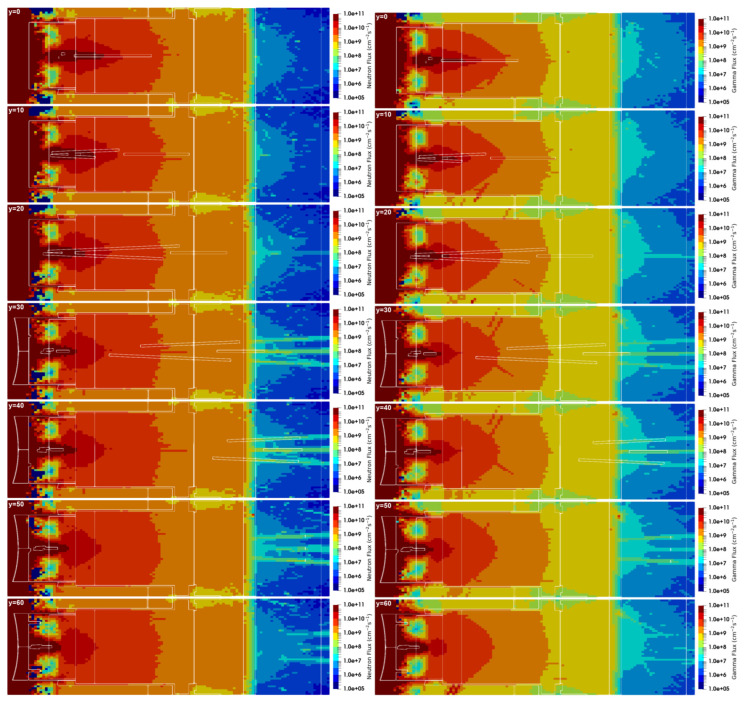
Neutron (n cm^−2^ s^−1^) and gamma (γ cm^−2^ s^−1^) fluxes in plane y with the alternative X-ray spectroscopy ducts.

**Figure 40:**
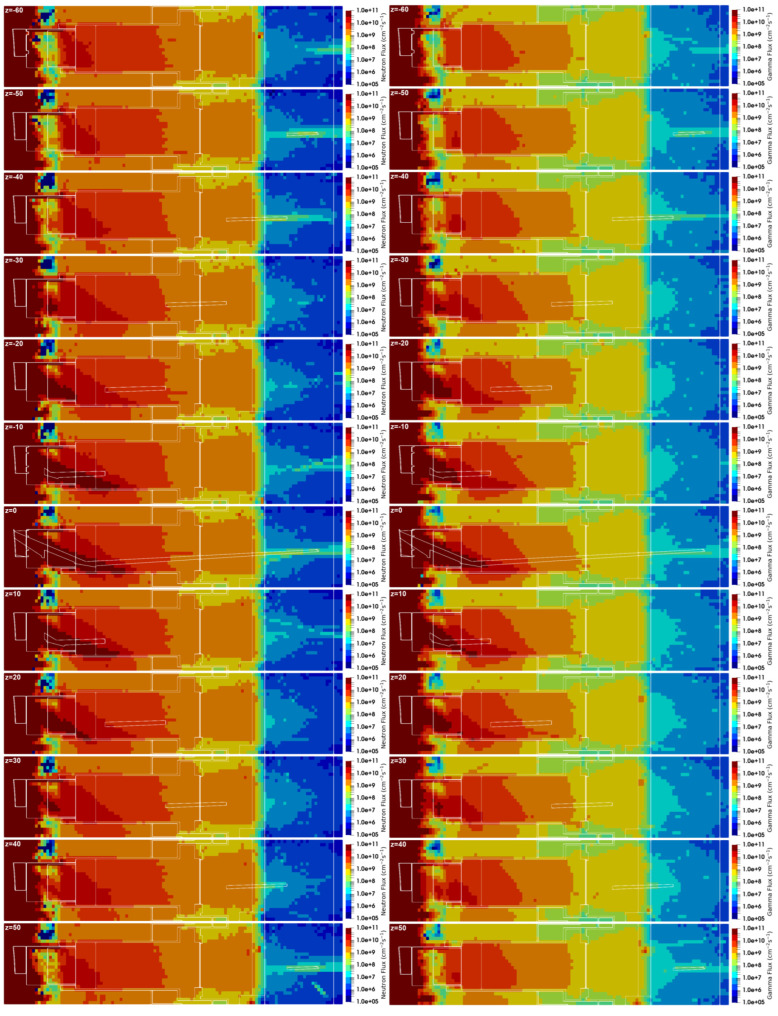
Neutron (n cm^−2^ s^−1^) and gamma (γ cm^−2^ s^−1^) fluxes in plane z with the alternative X-ray spectroscopy ducts.

The neutron and gamma fluxes obtained with the alternative duct configuration are presented in Figure 39 and Figure 40, for planes y and z. The neutron fluxes in the three crystal Bragg reflectors of the port cell were 5.6 × 10^7^, 1.2 × 10^8^ and 7.4 × 10^7^ n cm^−2^ s^−1^, with statistical errors of 24%, 43% and 10%, respectively. Even though 2 × 10^10^ particles were simulated, for 5–7 days per simulation and with 720 processors per simulation, the statistical errors are very large in all but one of the mirrors. Nevertheless, the results indicate that with a configuration like this, and including some shielding optimization, it should be possible to reduce the neutron fluxes to below 1 × 10^8^ n cm^−2^ s^−1^, which means a reduction by more than two orders of magnitude when compared to the straight ducts.

Similar reductions were obtained in the gamma fluxes: 2.6 × 10^7^, 6.2 × 10^7^ and 4.3 × 10^7^ γ cm^−2^ s^−1^ in the three mirrors, with statistical errors of 7%, 9% and 6%. Again, this is almost two orders of magnitude lower than the gamma fluxes obtained with the straight ducts.

As mentioned before, there are no limits defined for the neutron and gamma fluxes in the DEMO port cell, and no calculations of shutdown dose rates are provided here. However, it seems feasible, based on the simulation experience from ITER (although the dose rates will depend on the components present in the port cell and on the integrated fluxes) [62], to comply with the limit of 100 μSv/h in the port cell 12 days after shutdown with neutron fluxes below 1 × 10^8^ n cm^−2^ s^−1^ reaching the port cell. Nevertheless, shutdown dose rate simulations with models of the port cell components are required to assess the compliance with the limit.

It should also be mentioned that without further shielding, the alternative duct configuration presented here is expected to increase the nuclear heat loads in the toroidal field coils. Further studies are therefore required to calculate these loads and to compare the results between configurations.

## 4. Conclusions

This paper aimed to provide a broad view of the radiation environment that diagnostics in DEMO are expected to face, assuming as a reference the water-cooled lithium lead blanket (WCLL) configuration. Resorting to diagnostics representative of different integration approaches in DEMO—inner vessel, ex-vessel and equatorial ports—neutronics simulations were performed to estimate the fluxes, heat loads, dose rates and dpa in different sections of the tokamak, using pre-conceptual CAD models of the diagnostics.

The first simulations were related to inner-vessel diagnostics, distributed poloidally around the plasma: in-vessel magnetics sensors and the diagnostics slim cassette (DSC), projected for the integration of microwave reflectometry and the ECE. These simulations have shown that the introduction of the DSC designed for reflectometry will not compromise the mechanical integrity of the VV, as the fluxes and loads behind the DSC are comparable to the ones obtained behind the WCLL breeding blanket (BB) without the DSC. Another conclusion of this study is that the fluences in the magnetics sensors behind the blankets, integrated over the whole DEMO lifetime, are comparable to the ones expected for ITER, even though ITER will operate only for 0.54 FPY instead of the 6 FPY of DEMO. This is due to the excellent shielding performance of the current WCLL blanket design. It should be noticed, however, that the analysis presented here is not conservative: with the alternative helium cooled pebble bed (HCPB) blanket, the fluences in the sensors would increase up to a factor of 10, increasing the loads significantly in the in-vessel magnetics sensors in comparison with ITER. Additionally, the current WCLL blanket design is far from final, and a reduction in its shielding capability in the near future cannot be ruled out at this stage. Another important point is that the fluences below the divertor in DEMO are increased by at least a factor of 4 when compared to ITER. For all these reasons, R&D studies for magnetics sensors should still be based on the assumption that the loads in DEMO will exceed those expected for ITER.

Ex-vessel Faraday sensors were simulated next. As in ITER, this diagnostic is planned to be installed on the outer surface of the VV, with the aim to provide information on the plasma current during long plasma discharges. A maximum dose rate of 2.2 MGy/FPY was obtained in the simulations, which, integrated over 6 FPY, would exceed the 10 MGy considered as a conservative upper limit for the sensors’ lifetime dose in ITER. Nevertheless, this happens only in the divertor region, which is not modeled as accurately as the blanket in these simulations (and where the shielding has not been optimized yet). In the remaining regions, the dose rates are up to three orders of magnitude lower.

Finally, an equatorial port containing three diagnostics—X-ray spectroscopy, divertor monitoring and pellet monitoring—was simulated in detail. Considerable neutron streaming to the port cell was predicted with the initial design of the X-ray spectroscopy diagnostic, which foresaw large (10 cm × 23 cm) straight ducts between the plasma and the port cell. With neutron fluxes up to 2 × 10^10^ n cm^−2^ s^−1^, it can be anticipated that the current design would not comply with the dose rate limit of 100 μSv/h in the port cell 12 days after shutdown. A sensitivity analysis was then performed to evaluate the neutron streaming as a function of the duct cross-section, for diagnostics that require direct views of the plasma, without mirrors or doglegs. This study was extended to two such diagnostics, with 1 cm radius ducts: the neutron/gamma cameras and the radiated power and soft X-ray intensity diagnostic. Neutron fluxes of the order of 1–2 × 10^8^ n cm^−2^ s^−1^ were obtained in the port cell for those diagnostics. Finally, an alternative design of the X-ray spectroscopy diagnostic, based on graphite pre-reflectors, was shown to reduce the neutron fluxes in the port cell to ~1× 10^8^ n cm^−2^ s^−1^. This configuration might, however, increase the nuclear heat loads in the toroidal field coils. Accurate shutdown dose rate calculations in the port cell should be carried out in future work, along with a detailed study of the effect of the diagnostic port configurations on the nuclear heat loads in the magnets.

## Figures and Tables

**Table 1:** Neutron fluxes, nuclear heat loads and dose rates at 3 toroidal positions in the equatorial plane IB. For the flux calculations, the statistical errors are also presented. For the heat loads and dose rates, the neutron contribution to the total is included (remaining contribution by gammas).

Position			Between BBs (y = 0)	Behind DSC/BB (y = 10.5 cm)	Behind WCLL BB (y = 45 cm)
with DSC	Neutron flux (n cm^−2^ s^−1^)		4.56 × 10^12^ (1.8%)	1.22 × 10^12^ (3.6%)	7.38 × 10^11^ (4.7%)
	Gamma Flux (γ cm^−2^ s^−1^)		1.35× 10^12^ (3.1%)	4.72 × 10^11^ (5.3%)	7.34 × 10^10^ (13.9%)
	Nuclear heat load (mW cm^−3^)	Al_2_O_3_	82.84 (66%n)	11.60 (17%n)	3.14 (44%n)
		DP-951	65.79 (66%n)	8.86 (12%n)	2.17 (35%n)
		AlN	73.96 (69%n)	9.96 (21%n)	2.66 (46%n)
		MgO	82.86 (69%n)	10.89 (20%n)	3.04 (47%n)
		SiO_2_	62.55 (70%n)	8.13 (21%n)	2.33 (49%n)
		Si_3_N_4_	90.12 (70%n)	10.16 (23%n)	2.75 (47%n)
	Dose rate (Gy s^−1^)	Al_2_O_3_	20.97 (66%n)	2.94 (17%n)	0.79 (44%n)
		DP-951	21.22 (66%n)	2.86 (12%n)	0.70 (35%n)
		AlN	22.69 (69%n)	3.05 (21%n)	0.82 (46%n)
		MgO	23.15 (69%n)	3.04 (20%n)	0.85 (47%n)
		SiO_2_	23.62 (70%n)	3.07 (21%n)	0.88 (49%n)
		Si_3_N_4_	28.43 (70%n)	3.20 (20%n)	0.87 (47%n)
without DSC	Neutron flux (n cm^−2^ s^−1^)		4.96 × 10^12^ (1.8%)	1.41 × 10^12^ (3.4%)	8.12 × 10^11^ (4.5%)
	Gamma Flux (γ cm^−2^ s^−1^)		8.15× 10^11^ (3.9%)	1.98 × 10^11^ (8.4%)	7.68 × 10^10^ (13.4%)
	Nuclear heat load (mW cm^−3^)	Al_2_O_3_	76.97 (77%n)	7.43 (43%n)	3.41 (48%n)
		DP-951	60.47 (77%n)	5.24 (35%n)	2.30 (38%n)
		AlN	68.87 (80%n)	6.26 (45%n)	2.88 (50%n)
		MgO	77.99 (80%n)	7.28 (47%n)	3.31 (51%n)
		SiO_2_	59.24 (80%n)	5.54 (49%n)	2.55 (53%n)
		Si_3_N_4_	86.10 (84%n)	6.57 (47%n)	2.98 (51%n)
	Dose rate (Gy s^−1^)	Al_2_O_3_	19.49 (77%n)	1.88 (43%n)	0.86 (48%n)
		DP-951	19.51 (77%n)	1.69 (35%n)	0.74 (38%n)
		AlN	21.13 (80%n)	1.92 (45%n)	0.88 (50%n)
		MgO	21.79 (80%n)	2.03 (47%n)	0.92 (51%n)
		SiO_2_	22.37 (80%n)	2.09 (49%n)	0.96 (53%n)
		Si_3_N_4_	27.16 (84%n)	2.07 (47%n)	0.94 (51%n)

**Table 2:** Total heat loads in six materials at 60 poloidal locations behind the WCLL blanket.

Pos	Total Heat Loads (mW cm^−3^)												
	with DSC						without DSC						
	Al_2_O_3_	DP-951	AlN	MgO	SiO_2_	Si_3_N_4_	Al_2_O_3_	DP-951	AlN	MgO	SiO_2_	Si_3_N_4_	
1	165.4	131.0	143.6	157.1	116.6	152.6	165.8	131.3	143.9	157.5	116.9	152.9	Divertor
2	126.5	100.1	109.9	119.5	88.7	115.7	126.8	100.4	110.2	119.9	88.9	116.0	
3	49.12	38.73	42.43	45.89	34.02	43.55	48.80	38.45	42.16	45.61	33.81	43.27	
4	16.91	13.26	14.52	15.65	11.62	14.74	16.73	13.13	14.40	15.48	11.49	14.61	
5	9.03	7.09	7.67	8.40	6.23	7.78	8.73	6.83	7.42	8.15	6.04	7.53	
6	0.89	0.68	0.78	0.83	0.62	0.80	0.75	0.52	0.62	0.71	0.54	0.64	
7	1.27	0.98	1.10	1.18	0.88	1.11	0.57	0.43	0.49	0.54	0.41	0.50	
8	1.77	1.36	1.54	1.66	1.23	1.56	1.16	0.85	1.00	1.13	0.85	1.04	
9	1.84	1.38	1.61	1.74	1.31	1.65	1.65	1.19	1.36	1.60	1.21	1.43	
10	3.19	2.41	2.74	2.98	2.23	2.78	2.23	1.57	1.86	2.14	1.63	1.91	
11	5.13	3.87	4.36	4.80	3.60	4.42	3.37	2.34	2.81	3.28	2.50	2.89	
12	8.02	6.02	6.77	7.59	5.70	6.98	5.50	3.85	4.60	5.29	4.04	4.75	Eq. plane IB
13	10.66	8.14	9.08	10.05	7.50	9.32	5.95	4.10	4.95	5.77	4.44	5.21	
14	10.28	7.79	8.72	9.67	7.24	8.89	6.71	4.66	5.71	6.56	5.02	6.01	
15	9.49	7.17	8.21	8.96	6.71	8.40	7.09	4.97	6.09	6.92	5.27	6.32	
16	10.22	7.69	8.79	9.60	7.21	9.02	6.68	4.70	5.65	6.43	4.93	5.95	
17	6.52	4.95	5.57	6.10	4.57	5.68	4.96	3.42	4.13	4.77	3.66	4.24	
18	4.28	3.23	3.64	4.02	3.01	3.68	3.10	2.15	2.60	3.00	2.31	2.74	
19	3.80	2.88	3.21	3.55	2.65	3.24	2.35	1.64	1.94	2.33	1.78	2.08	
20	2.07	1.56	1.76	1.92	1.44	1.77	1.39	0.96	1.15	1.38	1.05	1.21	
21	1.28	0.96	1.07	1.20	0.90	1.08	0.71	0.50	0.63	0.69	0.53	0.63	
22	1.17	0.87	0.98	1.09	0.83	1.00	0.69	0.46	0.59	0.66	0.51	0.62	
23	1.37	1.01	1.11	1.30	0.98	1.15	0.75	0.52	0.63	0.71	0.55	0.64	
24	1.79	1.37	1.50	1.69	1.26	1.55	1.12	0.83	0.95	1.09	0.82	1.02	
25	1.59	1.21	1.36	1.49	1.11	1.35	1.21	0.85	1.03	1.19	0.90	1.05	
26	1.51	1.09	1.27	1.42	1.08	1.29	1.05	0.72	0.88	1.02	0.78	0.88	
27	2.90	2.28	2.52	2.68	1.98	2.55	1.99	1.51	1.74	1.87	1.40	1.78	
28	2.59	2.02	2.30	2.43	1.81	2.38	1.52	1.16	1.37	1.46	1.09	1.42	
29	2.71	2.15	2.42	2.50	1.85	2.46	2.34	1.82	2.08	2.21	1.64	2.18	
30	4.98	3.96	4.37	4.58	3.38	4.44	2.82	2.16	2.49	2.64	1.98	2.55	
31	3.19	2.50	2.76	2.96	2.20	2.84	2.54	1.96	2.16	2.38	1.78	2.25	
32	3.42	2.71	3.00	3.14	2.33	3.06	2.57	1.97	2.21	2.39	1.79	2.27	
33	3.84	3.01	3.28	3.52	2.61	3.30	2.65	2.05	2.25	2.47	1.85	2.33	
34	3.69	2.90	3.29	3.43	2.54	3.35	2.65	2.03	2.38	2.51	1.87	2.46	
35	3.39	2.64	2.95	3.13	2.33	2.99	2.31	1.75	2.02	2.18	1.63	2.05	
36	3.21	2.52	2.81	2.97	2.20	2.84	2.36	1.79	2.04	2.22	1.66	2.10	
37	3.51	2.78	3.09	3.23	2.39	3.14	2.63	2.30	2.29	2.43	1.77	2.39	
38	3.88	3.04	3.37	3.61	2.68	3.44	2.67	2.02	2.32	2.52	1.88	2.36	
39	3.22	2.47	2.88	3.05	2.28	2.97	2.15	1.58	1.90	2.08	1.57	1.99	
40	3.02	2.27	2.53	2.85	2.14	2.59	2.28	1.64	1.95	2.21	1.67	2.02	
41	3.14	2.35	2.62	2.96	2.23	2.67	2.31	1.59	1.91	2.25	1.72	1.98	Eq. plane OB
42	2.85	2.15	2.42	2.70	2.03	2.51	1.90	1.31	1.56	1.86	1.41	1.62	
43	3.18	2.35	2.70	3.07	2.31	2.81	2.12	1.47	1.81	2.16	1.64	1.98	
44	2.95	2.16	2.50	2.76	2.09	2.53	2.09	1.40	1.72	2.04	1.57	1.76	
45	3.11	2.30	2.63	2.93	2.21	2.67	2.18	1.44	1.80	2.14	1.65	1.88	
46	3.69	2.80	3.17	3.48	2.61	3.27	3.23	2.28	2.74	3.14	2.41	2.94	
47	4.53	3.42	3.87	4.22	3.16	3.91	3.22	2.24	2.75	3.10	2.38	2.87	
48	5.14	3.91	4.36	4.77	3.57	4.44	3.84	2.76	3.25	3.62	2.76	3.33	
49	5.50	4.19	4.65	5.13	3.84	4.75	3.52	2.49	3.05	3.40	2.60	3.23	
50	4.63	3.53	3.96	4.30	3.21	4.01	2.95	2.14	2.48	2.86	2.17	2.59	
51	3.35	2.50	2.89	3.11	2.34	2.92	2.57	1.83	2.13	2.47	1.89	2.26	
52	3.15	2.38	2.70	2.97	2.22	2.75	2.04	1.45	1.73	1.97	1.50	1.79	
53	3.06	2.33	2.67	2.85	2.14	2.73	1.75	1.21	1.51	1.68	1.29	1.53	
54	1.72	1.29	1.45	1.59	1.20	1.45	1.06	0.75	0.89	1.03	0.78	0.91	
55	2.17	1.64	1.82	2.02	1.52	1.84	1.39	1.01	1.16	1.32	1.00	1.17	
56	3.70	2.77	3.19	3.49	2.63	3.28	3.20	2.39	2.70	3.07	2.32	2.87	
57	34.54	27.33	29.48	32.30	23.94	30.51	34.19	27.04	29.14	31.96	23.70	30.16	Divertor
58	193.9	154.6	169.0	186.2	138.3	184.0	192.9	153.7	168.2	185.3	137.6	183.1	
59	170.4	135.2	148.7	163.1	121.0	159.6	170.1	134.9	148.4	162.7	120.8	159.2	
60	167.9	133.2	145.6	159.6	118.4	154.6	168.1	133.4	145.8	159.9	118.6	154.8	

**Table 3:** Dose rates in six materials at 60 poloidal locations behind the WCLL blanket.

Pos	Dose Rate (Gy s^−1^)												
	with DSC						without DSC						
	Al_2_O_3_	DP-951	AlN	MgO	SiO_2_	Si_3_N_4_	Al_2_O_3_	DP-951	AlN	MgO	SiO_2_	Si_3_N_4_	
1	41.87	42.26	44.04	43.88	44.01	48.13	41.97	42.36	44.14	43.98	44.11	48.23	Divertor
2	32.01	32.28	33.70	33.38	33.46	36.48	32.11	32.38	33.80	33.48	33.56	36.58	
3	12.43	12.49	13.01	12.82	12.84	13.74	12.35	12.40	12.93	12.74	12.76	13.65	
4	4.28	4.28	4.45	4.37	4.38	4.65	4.24	4.24	4.42	4.32	4.34	4.61	
5	2.29	2.29	2.35	2.35	2.35	2.45	2.21	2.20	2.28	2.28	2.28	2.37	
6	0.22	0.22	0.24	0.23	0.23	0.25	0.19	0.17	0.19	0.20	0.21	0.20	
7	0.32	0.32	0.34	0.33	0.33	0.35	0.14	0.14	0.15	0.15	0.15	0.16	
8	0.45	0.44	0.47	0.46	0.47	0.49	0.29	0.27	0.31	0.32	0.32	0.33	
9	0.47	0.44	0.49	0.49	0.49	0.52	0.42	0.38	0.42	0.45	0.46	0.45	
10	0.81	0.78	0.84	0.83	0.84	0.88	0.56	0.51	0.57	0.60	0.61	0.60	
11	1.30	1.25	1.34	1.34	1.36	1.39	0.85	0.76	0.86	0.92	0.94	0.91	
12	2.03	1.94	2.08	2.12	2.15	2.20	1.39	1.24	1.41	1.48	1.53	1.50	Eq. plane
13	2.70	2.63	2.79	2.81	2.83	2.94	1.51	1.32	1.52	1.61	1.67	1.64	
14	2.60	2.51	2.68	2.70	2.73	2.80	1.70	1.50	1.75	1.83	1.90	1.90	
15	2.40	2.31	2.52	2.50	2.53	2.65	1.79	1.60	1.87	1.93	1.99	1.99	
16	2.59	2.48	2.70	2.68	2.72	2.84	1.69	1.52	1.73	1.80	1.86	1.88	
17	1.65	1.60	1.71	1.70	1.73	1.79	1.25	1.10	1.27	1.33	1.38	1.34	
18	1.08	1.04	1.12	1.12	1.14	1.16	0.79	0.69	0.80	0.84	0.87	0.87	
19	0.96	0.93	0.99	0.99	1.00	1.02	0.60	0.53	0.59	0.65	0.67	0.66	
20	0.52	0.50	0.54	0.54	0.54	0.56	0.35	0.31	0.35	0.39	0.40	0.38	
21	0.32	0.31	0.33	0.34	0.34	0.34	0.18	0.16	0.19	0.19	0.20	0.20	
22	0.30	0.28	0.30	0.31	0.31	0.32	0.18	0.15	0.18	0.18	0.19	0.19	
23	0.35	0.33	0.34	0.36	0.37	0.36	0.19	0.17	0.19	0.20	0.21	0.20	
24	0.45	0.44	0.46	0.47	0.48	0.49	0.28	0.27	0.29	0.31	0.31	0.32	
25	0.40	0.39	0.42	0.42	0.42	0.43	0.31	0.27	0.32	0.33	0.34	0.33	
26	0.38	0.35	0.39	0.40	0.41	0.41	0.27	0.23	0.27	0.28	0.29	0.28	
27	0.73	0.73	0.77	0.75	0.75	0.80	0.50	0.49	0.54	0.52	0.53	0.56	
28	0.66	0.65	0.70	0.68	0.68	0.75	0.39	0.37	0.42	0.41	0.41	0.45	
29	0.69	0.69	0.74	0.70	0.70	0.77	0.59	0.59	0.64	0.62	0.62	0.69	
30	1.26	1.28	1.34	1.28	1.28	1.40	0.71	0.70	0.76	0.74	0.75	0.80	
31	0.81	0.81	0.85	0.83	0.83	0.90	0.64	0.63	0.66	0.66	0.67	0.71	
32	0.87	0.87	0.92	0.88	0.88	0.96	0.65	0.64	0.68	0.67	0.68	0.72	
33	0.97	0.97	1.01	0.98	0.98	1.04	0.67	0.66	0.69	0.69	0.70	0.73	
34	0.93	0.93	1.01	0.96	0.96	1.06	0.67	0.65	0.73	0.70	0.71	0.78	
35	0.86	0.85	0.90	0.87	0.88	0.94	0.59	0.56	0.62	0.61	0.61	0.65	
36	0.81	0.81	0.86	0.83	0.83	0.90	0.60	0.58	0.63	0.62	0.63	0.66	
37	0.89	0.90	0.95	0.90	0.90	0.99	0.67	0.74	0.70	0.68	0.67	0.75	
38	0.98	0.98	1.03	1.01	1.01	1.08	0.68	0.65	0.71	0.70	0.71	0.74	
39	0.82	0.80	0.88	0.85	0.86	0.94	0.54	0.51	0.58	0.58	0.59	0.63	
40	0.77	0.73	0.78	0.80	0.81	0.82	0.58	0.53	0.60	0.62	0.63	0.64	
41	0.79	0.76	0.80	0.83	0.84	0.84	0.58	0.51	0.59	0.63	0.65	0.62	Eq. plane
42	0.72	0.69	0.74	0.76	0.77	0.79	0.48	0.42	0.48	0.52	0.53	0.51	
43	0.81	0.76	0.83	0.86	0.87	0.89	0.54	0.48	0.56	0.60	0.62	0.63	
44	0.75	0.70	0.77	0.77	0.79	0.80	0.53	0.45	0.53	0.57	0.59	0.56	
45	0.79	0.74	0.81	0.82	0.83	0.84	0.55	0.47	0.55	0.60	0.62	0.59	
46	0.93	0.90	0.97	0.97	0.98	1.03	0.82	0.74	0.84	0.88	0.91	0.93	
47	1.15	1.10	1.19	1.18	1.19	1.23	0.81	0.72	0.84	0.86	0.90	0.91	
48	1.30	1.26	1.34	1.33	1.35	1.40	0.97	0.89	1.00	1.01	1.04	1.05	
49	1.39	1.35	1.43	1.43	1.45	1.50	0.89	0.80	0.94	0.95	0.98	1.02	
50	1.17	1.14	1.21	1.20	1.21	1.26	0.75	0.69	0.76	0.80	0.82	0.82	
51	0.85	0.81	0.89	0.87	0.88	0.92	0.65	0.59	0.65	0.69	0.71	0.71	
52	0.80	0.77	0.83	0.83	0.84	0.87	0.52	0.47	0.53	0.55	0.57	0.56	
53	0.77	0.75	0.82	0.80	0.81	0.86	0.44	0.39	0.46	0.47	0.49	0.48	
54	0.44	0.41	0.44	0.44	0.45	0.46	0.27	0.24	0.27	0.29	0.29	0.29	
55	0.55	0.53	0.56	0.57	0.57	0.58	0.35	0.32	0.36	0.37	0.38	0.37	
56	0.94	0.90	0.98	0.97	0.99	1.04	0.81	0.77	0.83	0.86	0.87	0.90	
57	8.74	8.82	9.04	9.02	9.03	9.62	8.66	8.72	8.94	8.93	8.94	9.51	Divertor
58	49.10	49.86	51.85	52.01	52.19	58.04	48.84	49.58	51.59	51.75	51.93	57.77	
59	43.13	43.60	45.60	45.55	45.67	50.33	43.05	43.51	45.52	45.45	45.57	50.23	
60	42.49	42.96	44.65	44.59	44.67	48.78	42.55	43.02	44.71	44.66	44.74	48.85	

**Table 4:** Neutron fluxes and fluences in the magnetics sensors (behind the WCLL blanket).

	Neutron Flux (n m^−2^ s^−1^)	Neutron Fluence (n m^−2^)			
		1 FPY	1.57 FPY	4.43 FPY	6 FPY
Blanket (min)	1.10 × 10^15^	3.47 × 10^22^	5.45 × 10^22^	1.54 × 10^23^	2.08 × 10^23^
Blanket (max)	1.40 × 10^16^	4.42 × 10^23^	6.94 × 10^23^	1.96 × 10^24^	2.65 × 10^24^
Divertor	5.20 × 10^16^	1.64 × 10^24^	2.58 × 10^24^	7.27 × 10^24^	9.85 × 10^24^

**Table 5:** Fluxes, heat loads and dose rates in the 72 positions of the FOCS (WCLL blanket).

Position	Neutron Flux (n/cm^2^/s)	Stat. Error (%)	Gamma Flux (γ/cm^2^/s)	Stat. Error (%)	Heat Load			Dose Rate (Gy/FPY)
					Total (W/cm^3^)	n (%)	γ (%)	
1	3.59 × 10^8^	3.0	1.25 × 10^8^	1.9	6.58 × 10^−7^	18.8	81.2	8.94 × 10^3^
2	2.92 × 10^8^	3.2	9.93 × 10^7^	1.9	5.27 × 10^−7^	23.0	77.0	7.17 × 10^3^
3	2.70 × 10^8^	3.1	9.07 × 10^7^	1.9	5.02 × 10^−7^	23.2	76.8	6.82 × 10^3^
4	2.32 × 10^8^	3.1	8.31 × 10^7^	2.0	4.57 × 10^−7^	25.3	74.7	6.22 × 10^3^
5	1.96 × 10^8^	3.3	7.02 × 10^7^	2.0	3.91 × 10^−7^	24.6	75.4	5.32 × 10^3^
6	1.79 × 10^8^	3.3	7.51 × 10^7^	1.9	3.96 × 10^−7^	22.8	77.2	5.39 × 10^3^
7	1.91 × 10^8^	3.1	8.82 × 10^7^	1.7	4.76 × 10^−7^	22.4	77.6	6.48 × 10^3^
8	2.74 × 10^8^	2.7	1.38 × 10^8^	1.4	7.05 × 10^−7^	21.9	78.1	9.59 × 10^3^
9	4.15 × 10^8^	2.3	2.08 × 10^8^	1.2	1.07 × 10^−6^	22.0	78.0	1.46 × 10^4^
10	5.65 × 10^8^	2.1	2.82 × 10^8^	1.1	1.53 × 10^−6^	23.3	76.7	2.08 × 10^4^
11	7.21 × 10^8^	1.9	3.70 × 10^8^	1.0	1.95 × 10^−6^	22.1	77.9	2.65 × 10^4^
12	8.69 × 10^8^	1.8	4.52 × 10^8^	0.9	2.40 × 10^−6^	21.7	78.3	3.26 × 10^4^
13	9.93 × 10^8^	1.7	5.26 × 10^8^	0.9	2.82 × 10^−6^	22.1	77.9	3.83 × 10^4^
14	1.08 × 10^9^	1.7	5.54 × 10^8^	0.9	2.94 × 10^−6^	21.9	78.1	4.00 × 10^4^
15	1.08 × 10^9^	1.7	5.69 × 10^8^	0.9	3.06 × 10^−6^	22.4	77.6	4.16 × 10^4^
16	1.09 × 10^9^	1.7	5.80 × 10^8^	0.9	3.11 × 10^−6^	22.1	77.9	4.23 × 10^4^
17	1.02 × 10^9^	1.7	5.59 × 10^8^	0.9	2.95 × 10^−6^	21.2	78.8	4.01 × 10^4^
18	9.74 × 10^8^	1.8	5.36 × 10^8^	0.9	2.93 × 10^−6^	22.2	77.8	3.98 × 10^4^
19	8.78 × 10^8^	1.9	4.62 × 10^8^	1.0	2.49 × 10^−6^	21.4	78.6	3.38 × 10^4^
20	7.23 × 10^8^	2.0	3.70 × 10^8^	1.1	1.97 × 10^−6^	22.3	77.7	2.68 × 10^4^
21	6.01 × 10^8^	2.2	2.91 × 10^8^	1.2	1.53 × 10^−6^	22.8	77.2	2.08 × 10^4^
22	4.56 × 10^8^	2.4	2.21 × 10^8^	1.3	1.14 × 10^−6^	20.7	79.3	1.55 × 10^4^
23	3.63 × 10^8^	2.6	1.64 × 10^8^	1.4	8.47 × 10^−7^	21.9	78.1	1.15 × 10^4^
24	3.40 × 10^8^	2.7	1.33 × 10^8^	1.6	6.97 × 10^−7^	23.0	77.0	9.48 × 10^3^
25	4.39 × 10^8^	2.3	1.36 × 10^8^	1.5	6.79 × 10^−7^	18.2	81.8	9.23 × 10^3^
26	1.10 × 10^9^	1.4	3.12 × 10^8^	0.9	1.63 × 10^−6^	16.5	83.5	2.21 × 10^4^
27	4.34 × 10^9^	0.8	9.42 × 10^8^	0.6	4.97 × 10^−6^	17.7	82.3	6.76 × 10^4^
28	1.70 × 10^10^	0.4	3.34 × 10^9^	0.4	1.84 × 10^−5^	22.1	77.9	2.50 × 10^5^
29	4.45 × 10^10^	0.3	8.98 × 10^9^	0.2	5.19 × 10^−5^	26.6	73.4	7.07 × 10^5^
30	8.16 × 10^10^	0.2	1.81 × 10^10^	0.2	1.08 × 10^−4^	28.4	71.6	1.47 × 10^6^
31	1.05 × 10^11^	0.2	2.27 × 10^10^	0.1	1.37 × 10^−4^	28.5	71.5	1.86 × 10^6^
32	1.31 × 10^11^	0.2	2.60 × 10^10^	0.1	1.63 × 10^−4^	29.3	70.7	2.21 × 10^6^
33	1.19 × 10^11^	0.2	2.28 × 10^10^	0.2	1.41 × 10^−4^	27.6	72.4	1.92 × 10^6^
34	9.15 × 10^10^	0.2	1.55 × 10^10^	0.2	9.85 × 10^−5^	29.0	71.0	1.34 × 10^6^
35	5.68 × 10^10^	0.3	8.62 × 10^9^	0.2	5.62 × 10^−5^	29.9	70.1	7.65 × 10^5^
36	3.35 × 10^10^	0.4	4.67 × 10^9^	0.4	3.13 × 10^−5^	30.8	69.2	4.26 × 10^5^
37	1.80 × 10^10^	0.7	2.60 × 10^9^	0.6	1.68 × 10^−5^	28.6	71.4	2.28 × 10^5^
38	1.24 × 10^10^	1.1	1.82 × 10^9^	1.0	1.15 × 10^−5^	28.6	71.4	1.56 × 10^5^
39	1.05 × 10^10^	1.3	1.57 × 10^9^	1.3	1.00 × 10^−5^	29.2	70.8	1.36 × 10^5^
40	8.93 × 10^9^	1.7	1.41 × 10^9^	1.4	8.78 × 10^−6^	27.5	72.5	1.19 × 10^5^
41	8.22 × 10^9^	2.1	1.33 × 10^9^	2.0	8.14 × 10^−6^	26.5	73.5	1.11 × 10^5^
42	7.31 × 10^9^	2.4	1.24 × 10^9^	2.6	7.44 × 10^−6^	25.5	74.5	1.01 × 10^5^
43	6.44 × 10^9^	2.6	1.17 × 10^9^	2.7	6.88 × 10^−6^	23.6	76.4	9.35 × 10^4^
44	6.10 × 10^9^	3.4	1.07 × 10^9^	2.9	6.08 × 10^−6^	23.9	76.1	8.27 × 10^4^
45	5.44 × 10^9^	3.1	9.86 × 10^8^	3.2	5.61 × 10^−6^	24.4	75.6	7.64 × 10^4^
46	5.21 × 10^9^	3.1	9.60 × 10^8^	3.2	5.56 × 10^−6^	23.4	76.6	7.56 × 10^4^
47	4.05 × 10^9^	4.5	8.56 × 10^8^	3.5	4.62 × 10^−6^	19.6	80.4	6.29 × 10^4^
48	3.69 × 10^9^	4.2	7.69 × 10^8^	4.0	4.01 × 10^−6^	18.7	81.3	5.45 × 10^4^
49	3.37 × 10^9^	4.9	6.66 × 10^8^	4.4	3.67 × 10^−6^	15.1	84.9	4.99 × 10^4^
50	3.12 × 10^9^	5.7	6.06 × 10^8^	5.2	3.13 × 10^−6^	17.1	82.9	4.26 × 10^4^
51	2.61 × 10^9^	5.1	5.53 × 10^8^	5.0	2.83 × 10^−6^	19.8	80.2	3.85 × 10^4^
52	2.32 × 10^9^	5.2	4.57 × 10^8^	4.2	2.26 × 10^−6^	14.8	85.2	3.08 × 10^4^
53	1.97 × 10^9^	4.8	3.83 × 10^8^	4.5	2.09 × 10^−6^	16.3	83.7	2.85 × 10^4^
54	1.97 × 10^9^	5.6	4.18 × 10^8^	17.9	2.16 × 10^−6^	15.9	84.1	2.94 × 10^4^
55	1.64 × 10^9^	7.8	3.04 × 10^8^	6.3	1.67 × 10^−6^	18.0	82.0	2.27 × 10^4^
56	1.45 × 10^9^	6.9	2.67 × 10^8^	6.0	1.41 × 10^−6^	17.3	82.7	1.92 × 10^4^
57	1.21 × 10^9^	7.5	2.11 × 10^8^	6.6	1.24 × 10^−6^	18.3	81.7	1.69 × 10^4^
58	1.09 × 10^9^	10.4	2.23 × 10^8^	6.7	1.06 × 10^−6^	16.4	83.6	1.44 × 10^4^
59	8.31 × 10^8^	8.1	1.61 × 10^8^	8.3	8.18 × 10^−7^	14.5	85.5	1.11 × 10^4^
60	5.59 × 10^8^	9.2	1.46 × 10^8^	6.0	7.17 × 10^−7^	10.4	89.6	9.76 × 10^3^
61	5.25 × 10^8^	8.9	1.16 × 10^8^	6.0	5.68 × 10^−7^	15.3	84.7	7.73 × 10^3^
62	5.01 × 10^8^	7.0	1.34 × 10^8^	5.2	6.83 × 10^−7^	12.9	87.1	9.30 × 10^3^
63	5.21 × 10^8^	7.0	1.22 × 10^8^	4.9	6.12 × 10^−7^	10.7	89.3	8.32 × 10^3^
64	3.60 × 10^8^	6.3	1.11 × 10^8^	5.6	5.57 × 10^−7^	10.8	89.2	7.58 × 10^3^
65	3.59 × 10^8^	5.7	1.01 × 10^8^	3.9	5.28 × 10^−7^	10.6	89.4	7.18 × 10^3^
66	3.73 × 10^8^	7.9	1.01 × 10^8^	3.5	5.03 × 10^−7^	12.0	88.0	6.84 × 10^3^
67	3.58 × 10^8^	4.7	1.05 × 10^8^	3.3	5.38 × 10^−7^	13.1	86.9	7.32 × 10^3^
68	4.25 × 10^8^	3.8	1.30 × 10^8^	2.4	6.38 × 10^−7^	17.7	82.3	8.68 × 10^3^
69	5.52 × 10^8^	3.3	1.78 × 10^8^	2.0	8.72 × 10^−7^	20.1	79.9	1.19 × 10^4^
70	9.79 × 10^8^	2.1	3.58 × 10^8^	1.2	1.78 × 10^−6^	23.1	76.9	2.42 × 10^4^
71	3.59 × 10^9^	0.9	2.23 × 10^9^	0.5	1.50 × 10^−5^	30.5	69.5	2.04 × 10^5^
72	1.53 × 10^9^	1.5	6.09 × 10^8^	0.9	3.50 × 10^−6^	27.6	72.4	4.76 × 10^4^

**Table 6:** Neutron fluxes as a function of the duct cross-sectional dimensions. BS refers to bioshield and M to a possible mirror location 2 m behind.

Neutron Flux (n cm^−2^ s^−1^)										
**Circular Ducts**	**Radius (cm)**	**Area** **(cm^2^)**	**Total BS** **(n cm^−2^ s^−1^)**	**Error** **(%)**	**Uncollided BS** **(n cm^−2^ s^−1^)**	**Error** **(%)**	**Total M** **(n cm^−2^ s^−1^)**	**Error** **(%)**	**Uncollided M** **(n cm^−2^ s^−1^)**	**Error** **(%)**
	1.5	7.1	5.38 × 10^8^	1.3	1.47 × 10^8^	0.5	3.68 × 10^8^	0.9	1.02 × 10^8^	0.6
	2	12.6	9.51 × 10^8^	0.7	2.63 × 10^8^	0.3	6.55 × 10^8^	0.7	1.82 × 10^8^	0.3
	2.5	19.6	1.50 × 10^9^	0.6	4.10 × 10^8^	0.2	1.03 × 10^9^	0.6	2.84 × 10^8^	0.2
	3	28.3	2.17 × 10^9^	0.5	5.90 × 10^8^	0.2	1.48 × 10^9^	0.5	4.09 × 10^8^	0.2
	3.5	38.5	2.97 × 10^9^	0.5	8.04 × 10^8^	0.1	2.02 × 10^9^	0.4	5.57 × 10^8^	0.2
	4	50.3	3.91 × 10^9^	0.5	1.05 × 10^9^	0.1	2.64 × 10^9^	0.4	7.26 × 10^8^	0.1
**Rectangular Ducts**	**Dimensions** **(cm × cm)**	**Area** **(cm^2^)**	**Total BS** **(n cm^−2^ s^−1^)**	**Error** **(%)**	**Uncollided BS** **(n cm^−2^ s^−1^)**	**Error** **(%)**	**Total M** **(n cm^−2^ s^−1^)**	**Error** **(%)**	**Uncollided M** **(n cm^−2^ s^−1^)**	**Error** **(%)**
	10 × 0.71	7.1	5.29 × 10^8^	0.6	1.47 × 10^8^	0.3	3.65 × 10^8^	0.6	1.02 × 10^8^	0.34
	10 × 1.26	12.6	9.71 × 10^8^	1.2	2.62 × 10^8^	0.2	6.60 × 10^8^	0.6	1.81 × 10^8^	0.26
	10 × 1.96	19.6	1.50 × 10^9^	0.9	4.10 × 10^8^	0.2	1.03 × 10^9^	0.5	2.84 × 10^8^	0.2
	10 × 2.83	28.3	2.18 × 10^9^	0.7	5.90 × 10^8^	0.2	1.49 × 10^9^	0.4	4.09 × 10^8^	0.17
	10 × 3.85	38.5	2.99 × 10^9^	0.8	8.03 × 10^8^	0.1	2.03 × 10^9^	0.4	5.56 × 10^8^	0.15
	10 × 5.03	50.3	3.86 × 10^9^	0.5	1.05 × 10^9^	0.1	2.63 × 10^9^	0.4	7.27 × 10^8^	0.13
	10 × 10	100	7.97 × 10^9^	1.0	2.09 × 10^9^	0.1	5.29 × 10^9^	0.3	1.45 × 10^9^	0.09
	10 × 15	150	1.20 × 10^10^	0.4	3.13 × 10^9^	0.1	7.91 × 10^9^	0.3	2.17 × 10^9^	0.07
	10 × 20	200	1.62 × 10^10^	0.4	4.17 × 10^9^	0.1	1.06 × 10^10^	0.2	2.89 × 10^9^	0.06
	10 × 23	230	1.90 × 10^10^	0.9	4.80 × 10^9^	0.1	1.22 × 10^10^	0.2	3.32 × 10^9^	0.06

**Table 7:** Gamma fluxes as a function of the duct cross-sectional dimensions. BS refers to bioshield and M to a possible mirror location 2 m behind.

Gamma Flux (γ cm^−2^ s^−1^)										
**Circular Ducts**	**Radius** **(cm)**	**Area** **(cm^2^)**	**Total BS** **(γ** **cm^−2^ s^−1^)**	**Error** **(%)**	**Uncollided BS** **(γ** **cm^−2^ s^−1^)**	**Error** **(%)**	**Total M** **(γ** **cm^−2^ s^−1^)**	**Error** **(%)**	**Uncollided M** **(γ** **cm^−2^ s^−1^)**	**Error** **(%)**
	1.5	7.1	7.50 × 10^7^	10.1	4.92 × 10^7^	9.5	4.48 × 10^7^	8.9	3.03 × 10^7^	9.1
	2	12.6	1.49 × 10^8^	9.3	8.09 × 10^7^	6.0	9.89 × 10^7^	10.6	5.47 × 10^7^	6.5
	2.5	19.6	2.06 × 10^8^	6.0	1.27 × 10^8^	5.0	1.25 × 10^8^	5.8	8.23 × 10^7^	5.4
	3	28.3	3.91 × 10^8^	9.8	2.08 × 10^8^	5.9	2.55 × 10^8^	10.8	1.37 × 10^8^	6.7
	3.5	38.5	5.03 × 10^8^	13.1	2.62 × 10^8^	4.6	3.32 × 10^8^	15.7	1.74 × 10^8^	5.2
	4	50.3	6.14 × 10^8^	4.4	3.55 × 10^8^	3.0	3.57 × 10^8^	5.0	2.24 × 10^8^	3.3
**Rectangular Ducts**	**Dimensions** **(cm × cm)**	**Area** **(cm^2^)**	**Total BS** **(γ** **cm^−2^ s^−1^)**	**Error** **(%)**	**Uncollided BS** **(γ** **cm^−2^ s^−1^)**	**Error** **(%)**	**Total M** **(γ** **cm^−2^ s^−1^)**	**Error** **(%)**	**Uncollided M** **(γ** **cm^−2^ s^−1^)**	**Error** **(%)**
	10 × 0.71	7.1	7.29 × 10^7^	8.78	4.77 × 10^7^	8.09	4.85 × 10^7^	9.74	3.30 × 10^7^	8.85
	10 × 1.26	12.6	1.61 × 10^8^	13.1	9.49 × 10^7^	6.85	1.01 × 10^8^	15.7	6.33 × 10^7^	7.44
	10 × 1.96	19.6	2.10 × 10^8^	8.3	1.38 × 10^8^	9.11	1.38 × 10^8^	9.34	9.30 × 10^7^	10.3
	10 × 2.83	28.3	3.41 × 10^8^	6.76	2.00 × 10^8^	4.32	2.23 × 10^8^	7.6	1.32 × 10^8^	4.2
	10 × 3.85	38.5	4.39 × 10^8^	5.57	2.66 × 10^8^	4.24	2.74 × 10^8^	6.35	1.75 × 10^8^	4.7
	10 × 5.03	50.3	6.14 × 10^8^	5.23	3.44 × 10^8^	3.95	3.80 × 10^8^	6.15	2.22 × 10^8^	4.58
	10 × 10	100	1.30 × 10^9^	4.63	7.27 × 10^8^	3.28	7.35 × 10^8^	5.91	4.44 × 10^8^	3.39
	10 × 15	150	2.17 × 10^9^	4.25	1.16 × 10^9^	2.46	1.20 × 10^9^	5.57	7.00 × 10^8^	2.91
	10 × 20	200	2.95 × 10^9^	2.31	1.60 × 10^9^	1.68	1.55 × 10^9^	2.9	9.32 × 10^8^	1.82
	10 × 23	230	3.47 × 10^9^	2.52	1.83 × 10^9^	1.8	1.83 × 10^9^	3.04	1.06 × 10^9^	1.8

**Table 8:** Summary of the neutron and gamma fluxes at the 13 detector positions of Figure 36.

Detector	Neutrons				Gammas	
	Total		Uncollided		Total	
	Flux (n/cm^2^/s)	Error (%)	Flux (n/cm^2^/s)	Error (%)	Flux (n/cm^2^/s)	Error (%)
1	1.07 × 10^8^	1.2	4.15 × 10^7^	0.3	1.94 × 10^7^	8.6
2	1.26 × 10^8^	1.1	5.07 × 10^7^	0.3	5.05 × 10^7^	46.4
3	1.41 × 10^8^	0.7	5.83 × 10^7^	0.2	2.78 × 10^7^	8.3
4	1.38 × 10^8^	0.7	5.89 × 10^7^	0.2	3.01 × 10^7^	13.5
5	1.54 × 10^8^	0.7	6.66 × 10^7^	0.2	3.18 × 10^7^	7.8
6	1.61 × 10^8^	0.6	6.94 × 10^7^	0.2	3.66 × 10^7^	9.2
7	1.60 × 10^8^	0.6	6.92 × 10^7^	0.2	3.65 × 10^7^	14.4
8	1.57 × 10^8^	0.7	6.70 × 10^7^	0.2	3.62 × 10^7^	9.6
9	1.52 × 10^8^	0.7	6.43 × 10^7^	0.2	3.08 × 10^7^	7.9
10	1.42 × 10^8^	0.8	5.95 × 10^7^	0.2	3.48 × 10^7^	13.5
11	1.33 × 10^8^	0.8	5.46 × 10^7^	0.3	3.16 × 10^7^	14.0
12	1.21 × 10^8^	1.0	4.66 × 10^7^	0.3	2.67 × 10^7^	18.3
13	1.04 × 10^8^	1.3	3.85 × 10^7^	0.4	2.23 × 10^7^	14.7

**Table 9:** Heat loads in Be by neutrons and gammas at the 13 detector locations of Figure 36.

Detector	Neutron				Gamma				Total	
	Total		Uncollided		Total		Uncollided			
	Heat Load (W/cm^3^)	Error (%)	Heat Load (W/cm^3^)	Error (%)	Heat Load (W/cm^3^)	Error (%)	Heat Load (W/cm^3^)	Error (%)	Heat Load (W/cm^3^)	Error (%)
1	3.02 × 10^−6^	0.4	2.62 × 10^−6^	0.4	1.94 × 10^−7^	11.4	1.38 × 10^−7^	9.4	3.21 × 10^−6^	0.5
2	3.68 × 10^−6^	0.3	3.22 × 10^−6^	0.3	2.33 × 10^−7^	9.4	1.73 × 10^−7^	7.4	3.91 × 10^−6^	0.4
3	4.18 × 10^−6^	0.4	3.65 × 10^−6^	0.3	2.34 × 10^−7^	10.3	1.69 × 10^−7^	6.8	4.41 × 10^−6^	0.5
4	4.21 × 10^−6^	0.3	3.70 × 10^−6^	0.3	2.59 × 10^−7^	7.5	2.02 × 10^−7^	7.2	4.47 × 10^−6^	0.3
5	4.77 × 10^−6^	0.3	4.19 × 10^−6^	0.2	2.59 × 10^−7^	6.2	2.02 × 10^−7^	5.7	5.03 × 10^−6^	0.4
6	4.97 × 10^−6^	0.2	4.38 × 10^−6^	0.2	3.12 × 10^−7^	8.1	2.16 × 10^−7^	5.5	5.28 × 10^−6^	0.3
7	4.96 × 10^−6^	0.2	4.37 × 10^−6^	0.2	2.91 × 10^−7^	6.7	2.16 × 10^−7^	5.5	5.25 × 10^−6^	0.3
8	4.83 × 10^−6^	0.3	4.25 × 10^−6^	0.2	2.68 × 10^−7^	7.0	2.16 × 10^−7^	6.3	5.10 × 10^−6^	0.4
9	4.67 × 10^−6^	0.4	4.10 × 10^−6^	0.3	2.91 × 10^−7^	8.0	2.13 × 10^−7^	5.7	4.96 × 10^−6^	0.5
10	4.29 × 10^−6^	0.4	3.75 × 10^−6^	0.3	3.01 × 10^−7^	12.1	2.03 × 10^−7^	6.9	4.59 × 10^−6^	0.5
11	3.94 × 10^−6^	0.3	3.45 × 10^−6^	0.3	2.36 × 10^−7^	8.7	1.69 × 10^−7^	7.5	4.17 × 10^−6^	0.4
12	3.37 × 10^−6^	0.4	2.93 × 10^−6^	0.4	2.21 × 10^−7^	9.7	1.72 × 10^−7^	8.7	3.59 × 10^−6^	0.5
13	2.84 × 10^−6^	0.5	2.45 × 10^−6^	0.4	1.55 × 10^−7^	7.9	1.35 × 10^−7^	8.6	2.99 × 10^−6^	0.7

## Data Availability

Not applicable.
